# Characterization of surface markers on extracellular vesicles isolated from lymphatic exudate from patients with breast cancer

**DOI:** 10.1186/s12885-021-08870-w

**Published:** 2022-01-10

**Authors:** Karin Ekström, Rossella Crescitelli, Hafsteinn Ingi Pétursson, Junko Johansson, Cecilia Lässer, Roger Olofsson Bagge

**Affiliations:** 1 Sahlgrenska Center for Cancer Research and Wallenberg Centre for Molecular and Translational Medicine, Department of Surgery, Institute of Clinical Sciences, Sahlgrenska Academy, University of Gothenburg, Gothenburg, Sweden; 2 Department of Surgery, Sahlgrenska University Hospital, Gothenburg, Sweden; 3 Krefting Research Centre, Department of Internal Medicine and Clinical Nutrition, Institute of Medicine, Sahlgrenska Academy, University of Gothenburg, Gothenburg, Sweden

**Keywords:** Extracellular vesicles, Exosomes, Breast cancer, Lymphatic drainage exudate, Flow cytometry, Multiplex phenotyping

## Abstract

**Background:**

Breast cancer is the most common cancer, and the leading cause of cancer-related deaths, among females world-wide. Recent research suggests that extracellular vesicles (EVs) play a major role in the development of breast cancer metastasis. Axillary lymph node dissection (ALND) is a procedure in patients with known lymph node metastases, and after surgery large amounts of serous fluid are produced from the axilla. The overall aim was to isolate and characterize EVs from axillary serous fluid, and more specifically to determine if potential breast cancer biomarkers could be identified.

**Methods:**

Lymphatic drain fluid was collected from 7 patients with breast cancer the day after ALND. EVs were isolated using size exclusion chromatography, quantified and detected by nanoparticle tracking analysis, electron microscopy, nano flow cytometry and western blot. The expression of 37 EV surface proteins was evaluated by flow cytometry using the MACSPlex Exosome kit.

**Results:**

Lymphatic drainage exudate retrieved after surgery from all 7 patients contained EVs. The isolated EVs were positive for the typical EV markers CD9, CD63, CD81 and Flotillin-1 while albumin was absent, indicating low contamination from blood proteins. In total, 24 different EV surface proteins were detected. Eleven of those proteins were detected in all patients, including the common EV markers CD9, CD63 and CD81, cancer-related markers CD24, CD29, CD44 and CD146, platelet markers CD41b, CD42a and CD62p as well as HLA-DR/DP/DQ. Furthermore, CD29 and CD146 were enriched in Her2+ patients compared to patients with Her2- tumors.

**Conclusions:**

Lymphatic drainage exudate retrieved from breast cancer patients after surgery contains EVs that can be isolated using SEC isolation. The EVs have several cancer-related markers including CD24, CD29, CD44 and CD146, proteins of potential interest as biomarkers as well as to increase the understanding of the mechanisms of cancer biology.

**Supplementary Information:**

The online version contains supplementary material available at 10.1186/s12885-021-08870-w.

## Background

Extracellular vesicles (EVs), such as exosomes and microvesicles, have been recognized to convey key messages in the molecular communication between cells. EVs have the capacity to shuttle proteins, lipids, and nucleotides such as RNA between cells, leading to an array of functional changes in the recipient cells [1]. Exosome secretion was first described in reticulocytes, and later, in other hematopoietic cells. Since the first findings, exosomes have also been shown to be produced by the cells of non-hematopoietic origin, such as epithelial cells, neurons, adipocytes and tumor cells [2]. They have also been identified in most bodily fluids including urine, amniotic fluid, blood, serum, saliva, ascites, breast milk, cerebrospinal fluid and nasal secretion [3]. EVs are involved in the communication between cells during normal physiology as well as in pathological conditions [4]. It is now being accepted that the secretion of EVs, such as exosomes, from cancer cells, has a profound impact on the initiation and propagation of metastatic breast cancer. Development of distant metastasis relies upon the generation of a permissive microenvironment in pre-metastatic niches, a process partly mediated by cancer-derived EVs that reprogram cells to support future entry of metastatic cells [5, 6].

Breast cancer is the most common diagnosed cancer and the leading cause of cancer-related deaths among women world-wide [7]. Axillary lymph node dissection (ALND) is a procedure in cancer patients with known lymph node metastases, where lymph nodes are removed to minimize the risk for further metastasis [8]. Large amounts of serous fluid are produced from the axilla post-operatively, and placing a drain in the axilla at the end of the surgery is a common practice to decrease post-operative seroma collection in the wound [9]. The amounts of fluid collected can vary widely, but can typically range between 20 and 300 ml during the first 24 h. That creates an opportunity to collect drain fluid from these patients for subsequent analysis and characterization of EVs. Liquid biopsies, and the analyses of EVs from cancer patients have the potential to improve the cancer diagnosis and treatment. Recently it was shown that exudative seroma from melanoma patients contain EVs enriched in proteins involved in melanoma progression, and that the content of EVs can be used to detect melanoma-specific mutations [10].

To our knowledge, no study has been performed to identify EVs in post-operative serous fluid from patients with breast cancer. The overall aim is to isolate and characterize EVs from axillary serous fluid, and more specifically to determine if potential biomarkers could be identified.

## Methods

All methods were carried out in accordance with relevant guidelines and regulations. The study was approved by the Swedish Ethical Review Authority (reference number 995–16) and written informed consent was obtained from all patients.

### Human sample collection

Lymphatic drain fluid was collected from the drain bags of breast cancer patients in the post-operative ward the day after ALND. Until collection, the drain fluid was stored in bags at room temperature (RT) and then transferred to the laboratory in 500 ml sterile bottles. Within 2 h after collection, lymphatic drainage fluid samples were centrifuged at 1880×*g* for 10 min followed by 2500×*g* for 10 min at RT to remove cells, debris and platelets. Samples were stored at − 80 °C until EV isolation.

### Isolation of extracellular vesicles by size exclusion chromatography

EVs were isolated from lymphatic drain fluid using qEV10/35 nm (Izon Science Ltd.) size exclusion chromatography (SEC) columns. The columns were prepared according to manufacturer’s protocol. Lymphatic drainage fluid samples stored at −80°C were thawed on water bath (~ 20 °C) and centrifuged at 3000×*g* for 10 min at RT to remove precipitates, cell debris and larger vesicles prior to loading on the column.

The isolation of EVs by qEV10/35 nm SEC was performed in two rounds: First, it was evaluated in which fractions most EVs were eluted, and secondly EV enriched fractions were isolated. To evaluate in which fractions EVs elute, lymphatic drainage fluid from 3 patients (patient 3, 5 and 6) was used. Ten ml fluid was overlaid on the column followed by elution with phosphate buffered saline (PBS). The void volume (20 ml) was discarded before collection of the individual fractions. Seven fractions of 5 ml were collected prior to further analysis. The 5 ml samples were concentrated using Amicon®ultra-4 ml 10 kDa centrifugal filters (Merck Millipore Ltd., Ireland) by centrifugation at 3200×*g* at 12 °C. Different fractions required different time for concentration, but for all samples, the time was kept a maximum of 1 h. The concentrated samples were transferred to new tubes, the volume was measured (between 500 and 700 μl) and samples were aliquoted and stored at − 80 °C until further analysis. To evaluate in which fractions most EVs elute, protein quantification, nanoparticle tracking analysis (NTA) and transmission electron microscopy was performed.

After it had been evaluated which fractions the EVs eluted in, a second round of isolations was performed. In this second step of SEC isolation, pooled EV enriched fractions (fraction 2–4) were isolated from all 7 breast cancer patients (patients 1–7). For this, 10 ml lymphatic drainage fluid was overlaid on the column, the first 25 ml were discarded (20 ml void volume and first 5 ml fraction) before collection of EV enriched pooled fractions 2–4 (15 ml). The EVs were concentrated using Amicon®ultra-15 ml 10 kDa centrifugal filters by centrifugation at 3200×*g* for maximum 1 h. The concentrated EVs were transferred to new tubes, the volume was measured before the EVs were aliquoted and stored at -80 °C.

### Nanoparticle tracking analysis (NTA)

To analyze which SEC fractions EVs were enriched in, the particle concentration of individual fractions (patient 3, 5 and 6, F1–7) was analyzed by Nanoparticle Tracking Analysis (NTA) using ZetaView® PMX120 (Particle Metrix, Meerbusch, Germany). Pooled EV fractions (F2-4) from patient 1–7 were also analysed using NTA. Polystyrene particles with a size of 100 nm (Merck) were used for instrument alignment. The samples were diluted with Dulbecco’s phosphate-buffered saline (dPBS, HyClone, 1:100–1:10000) to the optimal range for particle detection, loaded into the flow cell, and the instrument measured each sample at 11 different positions (3 cycles). For each sample, between 800 and 1800 particles were traced. The minimum brightness was 30, minimum size 10 and maximum size 1000.

After automated analysis of all frames, outlier positions were removed and the concentration was calculated by the ZetaView®software (version 8.05.12SP1). The instrument pre-acquisition parameters were: a sensitivity of 80, a frame duration of 1 s, and a shutter speed of 100. Post-acquisition parameters were set to a minimum brightness of 30, a minimum area of 10 and a maximum area of 1000 and trace length 15.

### Protein quantification and Western blot analysis

The protein concentration of the EVs (both individual fractions (F1–7) from patient 3, 5 and 6 and pooled EV fractions (F2-4) from patient 1–7) was measured using Qubit Protein Assay Kit in a Qubit 3 Fluorometer according to manufacturer’s protocol (ThermoFisher Scientific, Waltham, MA, USA). The protein extracts were loaded and separated on precast 4–20% polyacrylamide Mini-PROTEAN TGX gels (Bio-Rad Laboratories, Hercules, CA, USA). Pooled EV fractions (F 2–4): 10 μg protein extract was used for each patient except patient 2 where 9 μg was used due to low protein concentration. Individual fractions: 20 μg was used except for F1 in patient 4 and 6 where the maximum volume was loaded (2.5 and 3.4 μg respectively) due to limitation on protein. In order to visualize the proteins, the gels were then placed in a ChemiDoc MP Imaging System (Bio-Rad Laboratories) for activation by exposure to UV light (setting auto-exposure). Proteins were then transferred to Polyvinylidene fluoride (PVDF) membranes (Bio-Rad Laboratories). The membranes were blocked using Everyblot blocking buffer (Bio-Rad Laboratories) for 5 min and then incubated with primary antibodies at 4 °C overnight. All primary antibodies were diluted in Everyblot blocking buffer (Bio-Rad Laboratories). The primary antibodies used were anti-Grp94 (1:1000 dilution, clone 9G10, Enzo Life Sciences, Solna, Sweden), anti-Albumin (1:1000 dilution, clone EPR12774, Abcam Cambridge, UK), anti-CD63 (1:1000 dilution, clone H5C6, BD Biosciences), anti-flotillin-1 (1:1000 dilution, clone EPR6041, Abcam), anti-ApoA1 (1:500 dilution, GTX112692, GeneTex), anti-CD146 (dilution 1:1000, clone EPR3208, Abcam) and anti-EpCAM (1:1000, clone EPR20532–225, Abcam). To determine the CD63 expression the separation was performed under non-reducing conditions, for the other proteins, the separation was performed under reducing conditions. The membranes were washed three times in 1× Tris-buffered saline-Tween (TBST) and then incubated with the secondary antibody for 1 h at room temperature. The secondary antibody used for anti-flotillin-1, anti-ApoA1, anti-CD146, anti-Albumin and anti-EpCAM was anti-rabbit IgG (horseradish peroxidase conjugated, 1:5000 dilution, Harlan Sera-Lab, Loughborough, UK), the secondary antibody used for anti-CD63 was anti-mouse IgG (horseradish peroxidase conjugated, 1:5000 dilution, Harlan Sera-Lab) and the secondary antibody used for anti-Grp94 was anti-rat IgG (horseradish peroxidase conjugated, 1:5000 dilution, Harlan Sera-Lab). The secondary antibodies were diluted in Everyblot blocking buffer (Bio-Rad Laboratories). The membrane was then analyzed with the SuperSignal West Femto maximum sensitivity substrate (Thermo Fisher Scientific) and a ChemiDoc MP System (Bio-Rad Laboratories).

Imaging and data analysis was done in Image Lab™ Software (Bio-Rad Laboratories). CD146 was normalized and quantified by Stain-Free Total Protein Quantitation (background subtraction disc size 65) and the normalized volume intensity values were used for comparison between groups.

### Transmission electron microscopy (TEM)

Investigation of vesicles by negative staining was performed as previously described [11]. Briefly, 10 μg of vesicles was placed onto glow discharged 200-mesh formvar/carbon copper grids (Electron Microscopy Sciences, Hatfield Township, USA). After two washes in H_2_O, EVs were fixed in 2.5% glutaraldehyde and further washed two times in H_2_O. The samples were then stained with 2% uranyl acetate for 1.5 min. Negative-stained samples were investigated on a digitized LEO 912AB Omega electron microscope (Carl Zeiss SMT, Oberkochen, Germany) at 120 kV with a Veleta CCD camera (Olympus-SiS, Münster, Germany).

### Nano-flow cytometry (nFCM)

To analyze the size and concentration of isolated EVs (patient 1–7), EV samples were analyzed using the Flow Nano Analyzer (NanoFCM Inc.) according to manufacturer’s protocol. Briefly, 210 nm quality control beads (NanoFCM Inc.), were analyzed as a reference for particle concentration. Additionally, a cocktail of silica nanospheres (SiNPs) with diameters of 68, 91, 113 and 155 nm (NanoFCM Inc.) was analyzed to set reference for size distribution and PBS was analyzed as background signal. EV concentration and size distribution were calculated using the nFCM software (NanoFCM Profession V1.0). For estimation of particle size, a gate for 25–125 nm was used which comprised > 99% of the particles. Particle concentration values were converted to particles per ml of starting material.

### Multiplex EV surface marker analysis

Analysis of surface protein expression on EVs (patient 1–7) was performed using the MACSPlex Exosome kit human (Miltenyi Biotec, Bergisch-Gladbach, Germany) following the manufacturers protocol for overnight capture in tubes with few modifications. This kit enables the detection of 37 markers (CD1c, CD2, CD3, CD4, CD8, CD9, CD11c, CD14, CD19, CD20, CD24, CD25, CD29, CD31, CD40, CD41b, CD42a, CD44, CD45, CD49e, CD56, CD62p, CD63, CD69, CD81, CD86, CD105, CD133.1, CD142, CD146, CD209, CD326, HLA-ABC, HLA-DR DP DQ, MCSP, ROR1 and SSEA-4) simultaneously and include the two isotype controls (mIgG1 and REA control) corresponding to the antibodies. Briefly, 5 × 10^8^ EVs (quantified by nFCM) or PBS as blank control (both in triplicates) were diluted in 120 μl MACSPlex buffer. This corresponded to an EV protein amount of 7–29 μg. The EVs were incubated with 15 μl capture beads (containing the antibody-coated bead subsets) overnight at 4 °C under gentle agitation and protected from light. The EV-bead complexes were washed using 1 ml MACSPlex buffer and centrifuged at 3000 g for 5 min at RT. At all wash steps, 1 ml buffer was added and removed after the centrifugation step. Detection antibody mixture (CD9, CD63 and CD81 conjugated to APC) was added to the beads, samples were mixed by gentle vortexing and incubated for 1 h at RT under gentle agitation and protected from light. The samples were washed four times before analyzed on a BD FACSVerse™ Flow Cytometer running BD FACSuite™ software (BD Biosciences). Collected data were analyzed by FlowJo Software (tree Star Inc., Ashland, OR, USA).

Background values of PBS and the isotype controls (REA or mouse IgG) were subtracted from each of the sample PE median fluorescence intensity value (MFI) resulting in “background corrected CD9/CD63/CD81 PE MFI”. Mean values of the triplicate samples were calculated and background corrected CD9/CD63/CD81 PE MFI > 20 was considered as present. For all proteins with a background corrected CD9/CD63/CD81 MFI > 20 in any of the patients, a normalization was done for the technical triplicates based on mean MFI signals of CD9/CD63/CD81 resulting in “background corrected CD9/CD63/CD81 normalized MFI values” as described in manufacturers protocol. Background corrected CD9/CD63/CD81 MFI values were normalized to the mean signal of MFI for the CD9/CD63/CD81 beads within each sample resulting in background corrected CD9/CD63/CD81 normalized MFI values.

For principal component analysis (PCA), unsupervised hierarchical clustering and generation of a heatmap, the software Qlucore Omics Explorer (Qlucore, Lund, Sweden) was used. All proteins with a background corrected MFI > 20 in any of the patients were analyzed and normalized background corrected MFI values were used for the analysis. For PCA plots, the three technical replicates were included and plots with 1 and 3 nearest neighbors were created. Mean values of the three replicates were used for each patient EV sample to generate the unsupervised hierarchical clustering and the heatmap.

### Statistical analysis

The statistical analysis of differences in surface protein expression between two patient groups was performed with GraphPad Prism 9 Software (GraphPad Software Inc., La Jolla, CA, USA). Multiple t-tests with False Discovery Rate of 1% was used with a two-stage step-up method (Benjamini, Krieger and Yekuteli).

## Results

### EVs from lymphatic drainage fluid from breast cancer patients can be isolated by size exclusion chromatography

Lymphatic drainage fluid was collected from a total of 7 patients that underwent surgery due to breast cancer, one of the patients due to a recurrence of cancer in the axilla (patient 7). Two of the patients received neoadjuvant treatment before surgery (patients 1 and 4). The patient information is summarized in Table 1.

**Table 1:** Patient characteristics

Patient	Gender	Age	Breast cancer	NACT /Response#	Breast surgery	Axillary surgery	Histology	Tumor Size (mm)	NHG	ER+ (%)	PR+ (%)	HER2	LVI	Positive nodes
1	Female	72.4	Primary	Yes/Grade 2	Mastectomy	FNB- > ALND	Lobular	60	2	100	80	Neg	No	18
2	Female	86.1	Primary	No	Mastectomy	FNB- > ALND	Ductal	21	3	100	0	Neg	Yes	3
3	Female	40.5	Primary	No	Mastectomy	SNB- > ALND	Ductal	4	3	30	0	Pos	No	1
4	Female	45.3	Primary	Yes/Grade 5	BCS	FNB- > ALND	Ductal	0	3	15	0	Pos	No	0
5	Female	69.0	Primary	No	BCS	SNB- > ALND	Ductal	17	2	90	100	Neg	No	1
6	Female	70.0	Primary	No	Mastectomy	FNB- > ALND	Ductal	43	2	95	95	Pos	Yes	4
7	Female	66.3	Axillary recurrence	No	None	FNB- > ALND	Lobular	N/A	N/A	100	0	Neg	N/A	2

*ALND* axillary lymph node dissection, *BCS* Breast conserving surgery, *ER* estrogen receptor, *FNB* fine needle biopsy, *HER*2 human epidermal growth factor receptor 2, *LVI* lymphovascular invasion, *NACT* neoadjuvant chemotherapy, *NHG* Nottingham histological grade, *PR* progesterone receptor, *SNB* sentinel node biopsy. #according to Miller-Payne criteria for grading response.

To evaluate if lymphatic drainage fluid from breast cancer patients contains EVs, and if size-exclusion chromatography (SEC) is a suitable method for EV isolation, EVs from 3 breast cancer patients (patient 3, 5 and 6) were isolated using qEV SEC isolation, resulting in 7 fractions. In a first step prior to collecting 7 SEC fractions, 10 fractions were collected and analyzed from one patient, which showed that most EVs eluted at fraction 5 or earlier (Additional file 1). To evaluate in which of the 7 fractions EVs were eluted, each fraction was analyzed by protein quantification, nanoparticle tracking analysis (NTA), western blot and transmission electron microscopy (TEM) (Fig. 1). NTA and protein quantification of the individual fractions showed that EVs are mainly eluted in fraction 2–4, while proteins are eluted at later fractions (F5-7) (Fig. 1A). The ratio of EV number and protein amount can be used as an indication of EV purity [12]. In fraction 5–7, the EV number per protein amount was low compared to fraction 1–4, indicating protein contamination in fraction 5–7 (Fig. 1B).

**Figure:**
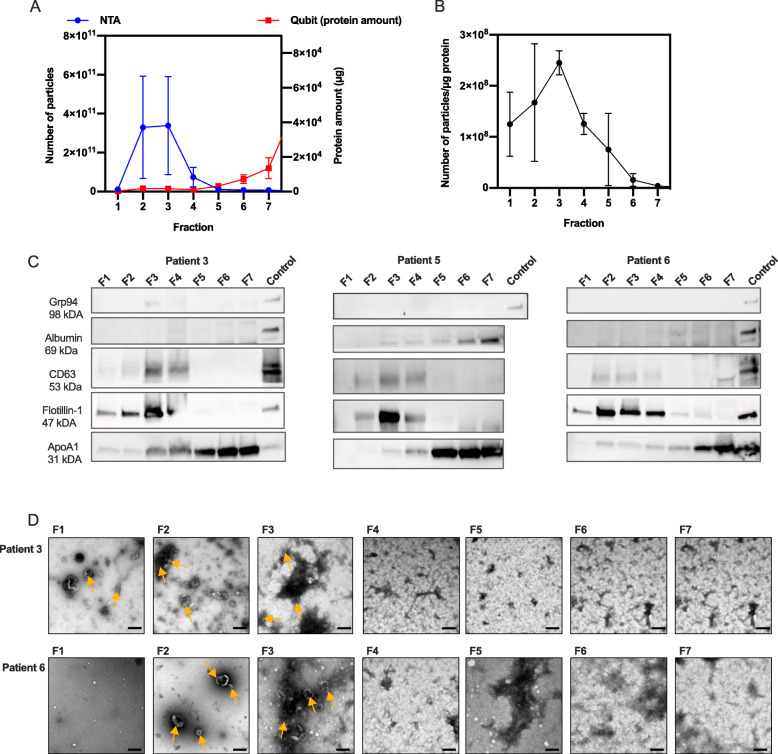
Evaluation of EV enriched fractions after SEC isolation of EVs from lymphatic drainage fluid from breast cancer patients. EVs were isolated from lymphatic drainage fluid of three patients (patient 3, 5 and 6) collected 1 day after breast surgery using qEV SEC. Seven fractions were collected, concentrated using ultrafiltration and analyzed. **A** Total particle number and protein amount and **B** particle to protein ratio in each of the 7 fractions analyzed by NTA and protein quantification by Qubit. **C** Fraction 1–7 (F1–7) were analyzed further by western blot (loading 20 μg except for F1 in patient 4 and 6 where the maximum volume was loaded (2.5 and 3.4 μg respectively) due to low protein concentration) detecting the EV markers Flotillin and CD63, endoplasmic reticulum marker Grp94, lipoprotein ApoA1 and albumin. EV proteins Flotillin and CD63 were mainly localized in F2–4, and non-EV proteins ApoA1 in F5–7. Albumin and Grp94 were not detected in any of the fractions. The positive control used was proteins extracted from MSC cell lysate (Grp94 and Flotillin-1), EVs from mesenchymal stem cells (CD63), human melanoma metastatic tissue (albumin and ApoA1). Western blot membranes are cropped, uncropped membranes are shown in Additional file 7. **D** TEM images of qEV fractions 1–7, patient 3 and 6. Size bar; 200 nm. Some of the vesicle-shaped particles are indicated by yellow arrows. EVs were mainly present in F1–3, while in F4–7, smaller, lipoprotein-like particles were covering most of the TEM grid. Values are mean values of the three patients and error bars indicates standard error of the mean

In order to evaluate the EV purity and protein contamination in the different fractions further, we used western blot to investigate typical EV markers, CD63 and Flotillin-1, as well as the non-EV proteins albumin and apolipoprotein A1 (Apo-A1) to evaluate blood contamination. The endoplasmic reticulum (ER) protein Grp94, which is normally not enriched in small EVs, was also included in the western blot antibody panel. This choice of markers was based on MISEV (2018) guidelines [13] (Fig. 1C). By western blot, it was shown that the typical EV markers CD63 and Flotillin-1 were mainly detected in fraction 2–4. For Flotillin-1, a slight band was also detected in fraction 1 for two of the patients. To further assess the purity of SEC fractions, we evaluated the presence of non-EV structures which are often co-isolated with EVs. In biofluids, EVs have been shown to co-isolate with lipoproteins, e.g., ApoA1 and albumin. Albumin and Grp94 were not detected in any of the fractions from any of the three patients. ApoA1 was mainly present in fraction 5–7 in all three patients. However, a slight band was also visible in F1–4 for patient 3, F3–4 for patient 5 and F2–4 for patient 6, indicating some contamination of lipoproteins in the EV-enriched fractions (Fig. 1C).

Electron microscopy analysis was performed to investigate EV structure and size. TEM images for patient 3 and 6 showed that structures with typical EV cup-shape were mainly present in F1–3, while in F4–7 not well-defined round structures were visible indicating presence of contaminants such as small, bright, lipoprotein-like structures visible mostly in fraction 5 from patient 6 (Fig. 1D).

Altogether, the combination of NTA, protein quantification, western blot and TEM, indicated that fractions 2–4 were EV-enriched with low contamination of non-EV elements, while non-EV proteins including lipoproteins were mainly eluted in the later fractions. Based on this, fraction 2–4 were isolated and pooled in the further experiments.

### Analysis of pooled EV fractions isolated from breast cancer patients show that small EVs are present in high numbers in all patients

**Fig. 2:**
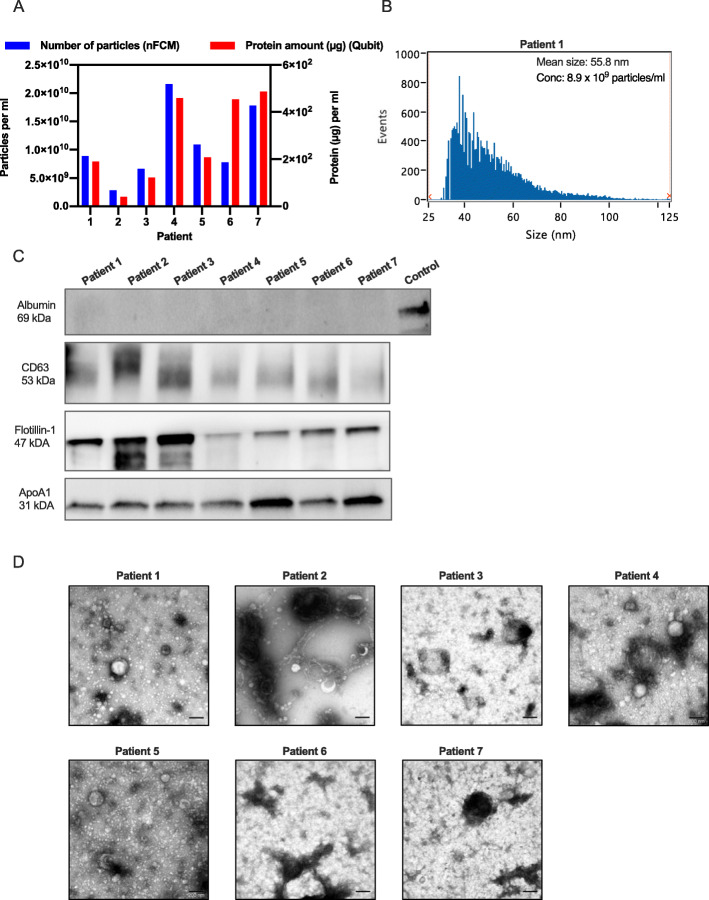
EVs isolated from lymphatic drainage fluid from breast cancer patients by SEC isolation. EVs were isolated from lymphatic drainage fluid of seven breast cancer patients using qEV SEC. EV-containing fractions were collected, pooled, concentrated using ultrafiltration and analyzed further. **A** EV particle number and protein amount isolated per ml of lymphatic drainage fluid from each of the 7 patients as analyzed by nFCM and Qubit. **B** Size distribution of EVs from patient 1 as analyzed by nFCM. **C** Western shows the presence of EV proteins CD63 and Flotillin and absence of albumin in EVs isolated from all patients. All EV samples contained lipoprotein ApoA1. The positive control used for albumin was human melanoma metastatic tissue and 9–10 μg protein was loaded in each lane. Western blot membranes are cropped, uncropped membranes are shown in Additional file 7. **D** TEM images show vesicle-like structure in the size range of 30–200 nm in EVs isolated from the breast cancer patients. Size bar, 200 nm

Using SEC isolation, EVs were isolated from the axillary lymphatic fluid of 7 breast cancer patients retrieved 1 day after surgery. EV enriched fractions (F2-4) were pooled and concentrated prior to analysis using nFCM, NTA, protein quantification, western blot and TEM (Fig. 2 and Additional file 2-4).

Nano-FCM data showed that from 10 ml of lymphatic drainage fluid, 1.1 × 10^11^ +/− 2.5 × 10^10^ particles were isolated (Fig. 2A). The protein amount correlated with the EV number for all patients except for patient 6, which contained a higher protein amount than predicted from the EV number (Fig. 2A). The particle to protein ratio, which can be used as an indication of EV purity, ranged between 1.7 × 10^7^ and 7.0 × 10^7^ particles per μg protein for the seven patients. Particle number was also quantified using NTA (Additional file 3A). By NTA, the particle number was estimated seven times higher compared to the quantification using nFCM (7.6 × 10^11^ +/− 1.9 × 10^11^ vs 1.1 × 10^11^ +/− 2.5 × 10^10^, Additional file 3B). Nano-FCM was also used to estimate the size of the EVs, based on the correlation to the size of beads with similar refractive index as EVs. The majority of EVs had a size between 30 and 100 nm, with a mean size around 50 nm (Fig. 2B and Additional file 4).

Using western blot, we showed that typical EV markers like CD63 and Flotillin-1 were present and Albumin was absent in EV samples from all 7 patients, indicating low contamination from blood proteins. However, all samples also contained ApoA1, indicating some contamination from lipoproteins. This finding, which is in line with the demonstration that ApoA1 is present in all qEV fractions (Fig. 1C), was supported by TEM, where it was shown that all EV isolations contain vesicle-like structures but also round, light elements which are likely to be lipoproteins (Fig. 2D). Taken together, these observations show that axillary lymphatic drainage fluid retrieved from breast cancer patients after surgery contains a high number of EVs, and those EVs can be isolated using qEV SEC isolation, even though lipoproteins cannot be eliminated completely.

### Surface protein profiling of EVs using multiplex protein analysis shows presence of cancer-related markers

**Fig. 3:**
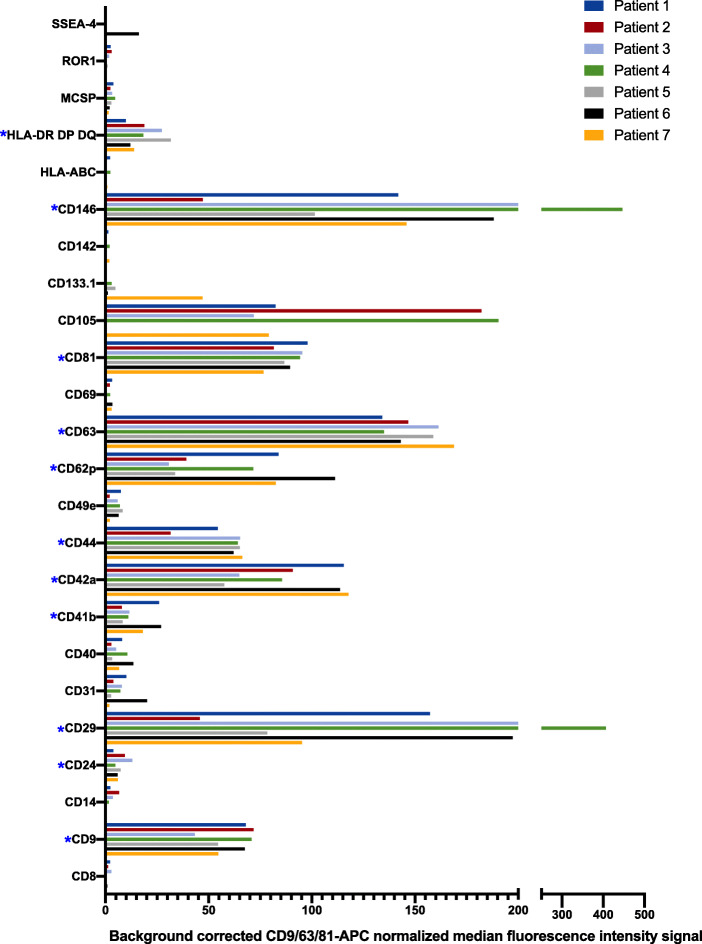
Surface marker profile of EVs isolated from lymphatic drainage fluid obtained from breast cancer patients. EV surface proteins were profiled using multiplex bead-based flow cytometry assay. Captured EVs were counterstained with APC-labeled detection antibodies (mixture of anti-CD9, anti-CD63, and anti-CD81 antibodies). The 24 out of 37 proteins that were found to be positive (MFI > 20) in any of the patients are shown in the figure. Data is presented as CD9/CD63/CD81 normalized background subtracted (isotype control and blank samples) median fluorescence intensity (MFI). Asterisks indicate proteins detected in all patients

In order to evaluate the expression of surface proteins on the EVs from breast cancer patients, the multiplex assay MACSPlex was performed, analyzing the signal of 37 surface proteins simultaneously. These proteins include both EV markers (CD9, CD63 and CD81), proteins that can indicate the cellular origin (e.g. CD3, CD4, CD8, CD14, CD41b, CD42a, CD45 and CD62p), cell activation markers (e.g. CD44, platelet activation marker CD62P (P-selectin)), antigen presenting proteins (HLA-DR/DP/DQ) and cancer-related markers (CD24, CD29, CD44 and CD146). The background corrected MFI of all markers is shown in Additional file 5. Twenty-four proteins were detected above the detection threshold in any of the patients. These proteins were normalized against the CD9/CD63/CD81 signal to minimize variation between different patients (Fig. 3). Eleven of those proteins were detected in all 7 patients (Table 2).

**Table 2:** Proteins detected in EVs from all 7 breast cancer patients

EV markers	Platelet markers	Cancer-related proteins	MHC class II
CD9	CD41b	CD24	HLA-DR
CD63	CD42a	CD29	HLA-DP
CD81	CD62p^a^	CD44	HLA-DQ
		CD146	

Proteins above the detection limit (background corrected MFI > 20) in all patients are shown in the table. ^a^activation marker.

Interestingly, four of those proteins (CD24, CD29, CD44 and CD146) have previously been associated with breast cancer [14–18]. In addition to the EV markers (CD9, CD63 and CD81), platelet associated-markers (CD41b and CD42a) and CD62p, which is expressed on activated platelets, endothelial cells and megakaryocytes were also detected on EVs from all patients, indicating that a portion of the EVs were of platelet origin. HLA-DR, HLA-DP and HLA-DQ was also present on EVs from all patients, indicating that some EVs were released from MHC class II containing cells, most probably antigen-presenting cells.

In order to analyze the MACSPlex data further, proteins that were present in most of the patients (at least 5 of the 7 patients) were evaluated (Table 3). Among these 16 proteins, proteins reflecting the cellular origin (HLA-DR, HLA-DP, HLA-DQ, CD31, CD41b, CD42a), activation markers (CD62p), EV markers (CD9, CD63, CD81), and cancer related markers (CD44 CD29, CD105, CD146) were found.

**Table 3:** Proteins detected by multiplex bead-based flow in EVs in at least 5 of 7 patients

Protein	Cellular expression	Extra information	Present in number of patients	Reference
*Proteins detected at high level, MFI > 400*				
CD9	Platelets, pre-B-cells, eosinophils, basophils, activated T-cells, endothelial and epithelial cells	EV marker, belonging to the tetraspanin family. It can modulate cell adhesion and migration, and is suggested to have a function in breast cancer metastasis.	7	[13, 21]
CD29 (Integrin beta 1)	Leukocytes, mesenchymal stem cells, cancer stem cells	Cell adhesion molecule and a marker of cancer stem cells. CD29 expression on EVs has been shown to be increased in breast cancer tissues.	7	[18, 24, 55]
CD42a	Platelets and megakaryocytes	Platelet marker. Platelets are suggested to be involved in all steps of tumorigenesis including tumor growth, tumor cell extravasation and metastasis. Platelet EVs have been shown to have a pro-coagulant function and be associated with aggressive tumors and poor prognosis.	7	[13, 56].
CD44	Various cells of different origins, e.g. cancer stem cells, hematopoetic cells and cells in the epidermis.	A cell adhesion molecule, receptor for e.g. hyaluronic acid. Highly expressed in many cancers. It has a role in cell migration, tumor growth and progression. It has been detected on EVs derived from plasma from breast cancer patients.	7	[14, 19, 25, 26]
CD62p (P-selectin)	Activated endothelial cells, platelets and megakaryocytes	Activation marker, expressed on activated endothelial cells and platelets. Associated with a high risk of venous thrombosis in cancer patients.	5	[27, 28]
CD63	Activated platelets, monocytes, macrophages, granulocytes, and endothelial cells	EV marker, belonging to the tetraspanin family.	7	[13]
CD81	B- and T-cells, NK cells, monocytes, thymocytes, DCs, endothelial cells, and fibroblasts	EV marker, belonging to the tetraspanin family.	7	[13]
CD105	Mature endothelial cells, mesenchymal stem cells, erythroid precursors, activated monocytes and macrophages. Plasma levels of soluble CD105 have been shown to correlate with metastasis in patients with breast cancer	Accessory receptor for transforming growth factor beta (TGF-β). Marker of cancer stem cells. It has a crucial role in angiogenesis, making it an important protein for tumor growth, survival and metastasis. Plasma levels of soluble CD105 have been shown to correlate with metastasis patients with breast cancer.	5	[17]
CD146 (MCAM)	Endothelial cells, pericytes, smooth muscle cells, follicular DC, melanoma cells, subpopulation of activated T-cells, marrow stromal cells (MSCs)	Cell adhesion molecule involved in the induction of epithelial-to-mesenchymal transition in breast cancer. Associated with high-grade tumors in breast cancer.	7	[16]
*Proteins detected at low level, MFI 20–400*				
CD24	B-cells, granulocytes, epithelial cells, monocytes, neuroblasts	Cell adhesion molecule, high levels in breast cancer tissue has been associated with poor prognosis.	7	[15]
CD31 (PECAM-1)	Monocytes, platelets, granulocytes, endothelial cells, lymphocyte subsets, and epithelial cells	Platelet endothelial cell adhesion molecule (PECAM-1). Used as marker of angiogenesis.	5	[29]
CD40	B-cells, monocytes, macrophages, follicular DCs, endothelial cells, fibroblasts, and keratinocytes	Costimulatory protein found on antigen-presenting cells and is required for their activation. High expression correlates with overall survival in various types of cancer.	5	[30]
CD41b	Platelets and megakaryocytes	Platelet marker, cell adhesion.	7	[31]
CD49e (Integrin alpha 5)	Thymocytes, T-cells, early activated B-cells, monocytes, platelets, fibroblasts, endothelial, and epithelial cells	Member of the integrin family. CD49e associates with CD29 (integrin β1 chain) to form the fibronectin receptor (Integrin α5β1). Integrin α5β1 has a role in carcinogenesis and cancer progression, and has been shown to be up-regulated in breast cancer cells, while functioning as tumor suppressors in some types of cancer and in cancer cell lines.	6	[32, 33]
MCSP (CSPG4)	Some cancer cells	Transmembrane proteoglycan. Expressed in breast cancer. High level in breast cancer tissue has been suggested to correlate with poor outcome.	6	[34, 61]
HLA-DR-DP-DQ	Antigen presenting cells and activated T cells	MHC class II, antigen presentation	7	

Cellular expression adapted from [61]

Some of the proteins included in the flow cytometry analysis were not detected in EVs from any of the patients (Additional file 5). Either they were not present on the EV surface, or below the detection limit. These proteins include epithelial cell adhesion molecule (EpCAM, CD326). The absence of EpCAM was also confirmed by western blot (Additional file 6.) Furthermore, the immunological related proteins (e.g. CD1C, CD2, CD3, CD4, CD11C, CD19, CD20, CD25, CD56, CD86, CD209) as well as the hematopoietic marker CD45 was not detected, indicating that the majority of EVs are not produced by immune cells.

In summary, the surface protein profile of EVs from breast cancer patients suggested that EVs with the typical EV markers CD9, CD63 and CD81 were of cancer cell origin, as well as released by antigen-presenting cells and platelets.

### Analysis of EV surface proteins reveal two main clusters related to tumor burden

**Fig. 4:**
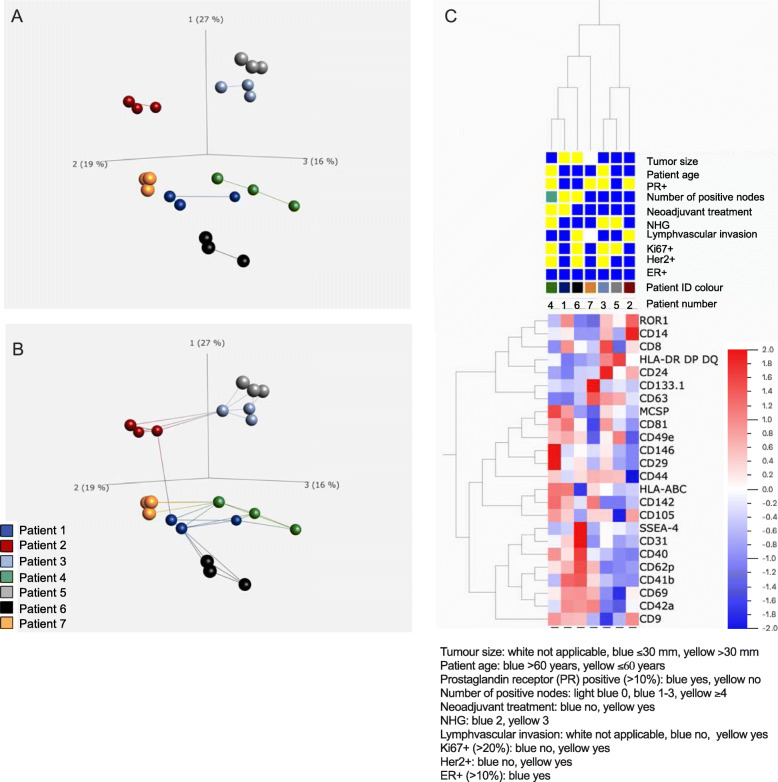
Principal component analysis (PCA) and hierarchical clustering heatmap of EV surface proteins detected by MACSPlex analysis. EV surface proteins detected by MACSPlex analysis in any of the patients were analyzed further using the bioinformatics software Qlucore. A-B) PCA illustrates the relationship between the technical replicates and variation between different patients were **A** shows connection between 1 nearest neighbor and **B** 3 nearest neighbors. **C** Hierarchical clustering heatmap of mean values for each patient. As illustrated by both PCA and heatmap, patient 2, 3, and 5 cluster together, and patient 1, 4, 6 and 7 cluster together

Cluster analysis was performed on the 24 EV surface proteins which were detected in any of the patients (Fig. 4). First, a principal component analysis (PCA) was performed, including all three replicates for each patient sample (Fig. 4A and B). As visualized in the PCA graphs, the technical replicates for each patient cluster together, indicating low variation between the replicate samples. Furthermore, the patients seem to spread into two main clusters separated by the principal component 1 explaining 27% of the differences. The first cluster included patient 2, 3 and 5, and the other included patient 1, 4, 6 and 7. Next, a hierarchical clustering heat-map was created including the normalized mean values for each protein (Fig. 4C). Also, in the heat-map, the two clusters (patient 2, 3, 5 and patient 1, 4, 6 7) were clearly visible. An interesting finding is that patient 7, having a lymph node recurrence, clustered closer to patient 1, 4 and 6. These patients had larger tumors, more lymph node metastasis and had received neoadjuvant chemotherapy (patient 1 and 4). It seemed to be no specific differences in clustering based on subtype or aggressiveness of the breast cancer (ER, PR, HER2+, Ki67 or grade).

### CD29 and CD146 are enriched on EVs from Her2 positive patients

**Fig. 5:**
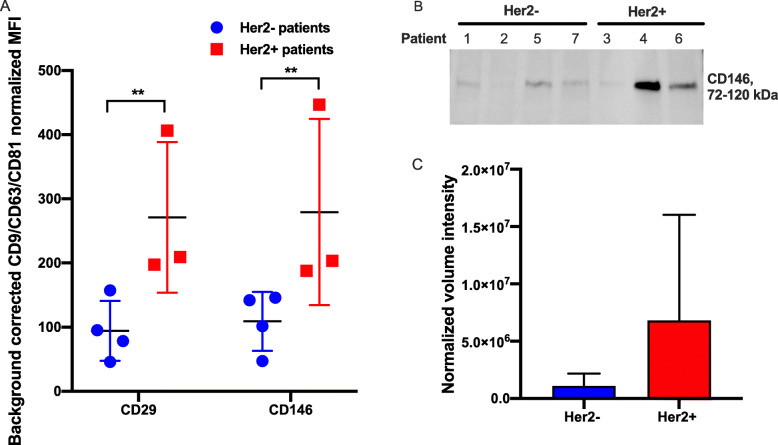
Comparison of surface protein expression in EVs from Her2 positive and Her2 negative patients show that CD29 and CD146 are increased in Her2 positive patients. **A** Background corrected CD9/CD63/CD81 normalized intensity signal for the surface proteins CD29 and CD146 that differed between Her2+ and Her2- patients in the MACSPlex analysis. (B-C) CD146 was further evaluated by western blot (10 μg protein was loaded in each lane except for patient 2 where 9 μg was used due to low protein concentration). **B** Western blot and **C** the normalized western blot quantification for CD146. Western blot membranes in the figure were cropped, uncropped membranes and the unstained gel used for the normalization are shown in Additional file 7. The Imaging and data analysis was done in Image Lab™ Software (Bio-Rad Laboratories). CD146 was normalized and quantified by Stain-Free Total Protein Quantitation and the normalized volume intensity values are shown in the figure. Results shown as mean values and standard deviation. **p < 0.01, q < 0.01

In an attempt to explore the results from the EV surface protein characterization further, we grouped the patients based on if they were positive or negative for Her2, and compared the expression of surface proteins on these EVs. These revealed that two of the proteins, CD29 and CD146 were enriched in EVs from Her2+ patients compared to EVs from Her2- patients (Fig. 5A). The increased expression of CD146 in Her2+ patients was validated by western blot, showing the same trend as the MACSPlex data (Fig. 5B-C). There was also a correlation between low age and high expression of CD29, even though the samples size was too small for any statistical analysis.

## Discussion

In this study, we demonstrated that exudate from lymphatic drainage retrieved from breast cancer patients after surgery contains EVs. Using SEC, we were able to isolate a high number of EVs from seven breast cancer patients. The yield of EVs from the lymphatic drainage fluid was in similar range as in plasma [36]. These EVs were positive for the common EV markers, as well as several cancer-related markers and platelet markers. Two distinct clusters, seemingly related to tumor burden, was identified, and EVs from Her2 positive patients were enriched in the cancer-related markers CD29 and CD146. EVs in lymphatic exudate fluid after surgery are easily accessible at sufficient number, which makes them attractive for the potential use as biomarkers. Furthermore, since EVs from cancer cells are believed to participate in most events of carcinogenesis and metastasis [37], these EVs may have a role in these events. It has been shown in a melanoma model that tumor derived EVs homed to sentinel lymph nodes, affecting cancer cell recruitment, extracellular matrix, and vascular proliferation within these lymph nodes [38]. To further shed light on the role of EVs in the development of lymphatic metastasis, the current study gives valuable information on how to analyze EVs derived directly from the lymphatic from patients.

To the best of our knowledge, this is the first report describing the isolation and surface-marker detection of EVs from lymphatic exudate from breast cancer patients.

EVs can be isolated using numerous different methods including sequential centrifugation, precipitation methods, density gradients and SEC [39]. The choice of isolation method depends on the source (e.g., cell culture medium, plasma, serum or tumor tissue), volume of starting material, as well as the planned downstream analysis. There is always a compromise between EV purity and yield as well as isolation time when isolating EVs. Furthermore, depending on the downstream analysis, it can be different demands on EV purity.

Common problems when isolating EVs from blood plasma and other biological fluids are co-isolation of albumin, lipoproteins and other non-EV proteins [40]. In a recent study, EV isolation from plasma using SEC and ultracentrifugation was compared. It was shown that SEC isolation resulted in higher yield and less contamination of albumin, but more lipoproteins compared to ultracentrifugation due to the overlap in size [40]. Similar to the blood plasma, lymphatic drainage fluid is a complex fluid which makes the isolation of pure EVs challenging. Since serum and lymphatic drainage fluid have many similarities, and SEC isolation have been shown to be suitable for isolation of serum-derived EVs [40, 41], we choose to use a commercial SEC column (qEV) for EV isolation in this study. These columns have previously been used to isolate EVs from different biofluids as well as cell culture media [42].

We used the qEV/35 nm column, which is supposed to mainly isolate small EVs (sEVs) less than 110 nm. To evaluate in which fractions the majority of EVs elute, we first collected 7 individual fractions from 3 different patients. These fractions were analyzed for particle number, protein amount, EV markers, non-EV markers/contamination markers as well as the morphology and size. According to the qEV instruction manual, most EVs are supposed to elute in fraction 1–4, with a main peak in fraction 2–3 for blood plasma sample. This is in line with our finding, where we showed that the majority of EVs, and EV related markers CD63 and Flotillin-1, are detected in fraction 2–4, while the protein content and non-EV proteins increased in fraction 5–6.

Even though fraction 1 displayed a weak band for Flotillin-1 in 2 out of 3 patients, and showed vesicle-like structures in TEM, this fraction contained very few EVs. Based on this we decided to isolate pooled fraction 2–4 as EV fraction throughout this study. The particle to protein ratio can be used as an indication of EV purity, and it has been suggested that a ratio of > 3 × 10^10^ particles/μg indicates pure EVs, while any ratios < 1.5 × 10^9^ indicates that the samples are unpure [12]. In this study the particle to protein ratio was used as an indication of which fractions contained most EVs with lowest contamination of proteins. The particle to protein ratio of EV enriched fractions 2–4 in this study were 1.6 × 10^8^, and the ratio was around 100 times lower in non-EV fractions 5–7, indicating that fraction 2–4 contained purest EVs. However, according to Webber and Clayton this would suggest that the EVs are unpure. The particle to protein ratio will depend on the method for quantification and will most likely vary between different laboratories. Interestingly, in another recent publication comparing several isolation methods including qEV, precipitation, ultracentrifugation and different density gradients, isolation by qEV combined with density gradient resulted in the highest purity followed by qEV alone, as estimated by the particle to proteins ratio (2 × 10^9^ and 5 × 10^8^ particles per μg protein) [41], which is in line with the current data.

A problem due to the overlapping size and possible co-isolation of lipoproteins and EVs, is that also lipoproteins will be counted using NTA or nFCM, potentially overestimating the number of EVs. However, according to the western blot and TEM results, the majority of lipoproteins were present in the non-EV fractions and thus they were removed during the EV isolation. In this study, the apolipoprotein A1 (ApoA1), which together with ApoA2 is the major constituent of high-density lipoprotein (HDL), was mainly distributed in fractions 5–7. However also fractions 1–4 contained ApoA1, which was also seen when isolating the pooled EV fraction 2–4. This indicates that even though the lipoprotein contamination is limited, only qEV SEC is not sufficient to isolate pure EVs from lymphatic drainage fluid without any contamination of lipoproteins. This is in line with previous studies showing that by using SEC, lipoproteins are co-isolated with EVs due to the overlap in size. However, the combination of SEC and density gradient can successfully separate EVs from most plasma proteins and lipoproteins since the density differs [43, 44]. Depending on the downstream analysis of EVs, the demands on EV purity and yield differ. The approach when evaluating the surface proteins in this study was to adhere EVs onto beads coated with different antibody and then counterstain with CD9/CD63/CD81 antibody. Using this approach, the proteins we detect will be on CD9, CD63 and/or CD81 positive particles, thus contaminating lipoproteins or other non-EV proteins will most likely not be a major problem since they will not interfere with our analysis of EV surface proteins.

EVs can be evaluated for particle number and size using a number of different methods, including Nanoparticle Tracking Analysis (NTA), tunable resisting pulse sensing (TRPS) as well as nano-flow cytometry (nFCM) [45]. Other methods are more suitable for evaluating the size but not particle number, e.g. electron microscopy, dynamic light scattering and atomic force microscopy (AFM) [39]. In this study, we used nFCM and NTA to quantify EVs, while we only used nFCM to evaluate size since nFCM has been shown to more accurately resolve the size of small particles [45]. Today there is no consensus in how to quantify EVs, and it is well-known that the particle number will depend on the quantification method used [46]. However, in a recent study evaluating EVs from human renal cancer tissue, quantification using NTA and nFCM resulted in similar particle number [47]. Even though we found a correlation between the methods, the EV number was 7-fold higher when quantifying with NTA compared to the nFCM. The EV number varied less than 10-fold between different patients. In this study, we cannot rule out whether this is a true biological variation in EV number, or due to variation in the EV isolation and quantification. Indeed, cancer cells release more EVs compared to non-cancer cells and EV number has been associated with tumor burden [48]. In this study, the number of patients were too few to make any conclusion about correlation between EV number and tumor burden, e.g. tumor size or positive lymph nodes and EV number.

EV surface proteins can potentially reflect the cellular origin and molecular pathology in different diseases, including breast cancer. In an attempt to evaluate the source of EVs in lymphatic drainage fluid from breast cancer patients, and to identify possible biomarkers, we evaluated the expression of 37 different surface proteins. Even though the patients included in this study had different tumor burden, the majority of the proteins were expressed at similar level in most of the patients. The EV markers CD9, CD63 and CD81 were expressed at high level in all the patients, with the highest expression of CD63, followed by CD81 and CD9. Since these markers were used for the normalization of MACSPlex data, the high and similar expression of these markers between different patients is of significance.

Interestingly, according to our MACSPlex results, the EVs do not express CD1C, CD2, CD3, CD4, CD11C, CD19, CD20, CD25, CD45, CD56, CD86, CD209 or CD326. However, since the detection of proteins is based on the co-localization with CD9, CD63, CD81, we cannot rule out the presence of EVs not expressing these markers even though this is very unlikely since at least CD9, CD63 or CD81 are believed to be present on most EVs [11]. In lymphatic drainage exudate, the majority of cells are immune cells, but cells and cell products of tumor origin have also been identified [49]. The finding that EVs do not contain CD45 is of special interest since it indicates that the EVs was not derived from hematopoietic cells, which also correlates with the absence of e.g. CD3. Furthermore, breast cancer cells as well as circulating tumor cells are of epithelial origin, and thus CD45 negative [50], it is thus possible that the EVs originate from those cells. One other possible explanation for the absence of CD45 on the EVs is that CD45 is mainly distributed on microvesicles and not on small EVs/exosomes, which are the main population of EVs that we studied [50, 51].

EpCAM (epithelial cell adhesion molecule, CD326) is expressed on normal epithelial cells, but is highly over-expressed in many types of cancer. The expression of EpCAM has been associated with cancer cell proliferation and metastasis, as well as with cancer stem cells [52]. EpCAM is released on EVs, and EpCAM positive EVs has been associated with different cancer diseases including breast and ovarian cancer. In ovarian cancer, the level of EpCAM positive EVs has been shown to correlate with severity of disease [53]. EpCAM, as well as the cancer-related protein CD24, has been detected on EVs isolated from ascites and pleural effusions from breast cancer patients. However, EVs isolated from the serum of those patients contained only CD24 and not EpCAM. Instead, EpCAM was found in a soluble form and not on EVs, which was shown to be due to metallo-proteinase (MMP) cleavage [54]. In this study, a low signal of CD24 was detected in all patients, while EpCAM was not detected in any of the patients. It is likely that similar cleavage occurs in the lymphatic drainage exudate as in the blood, since lymphatic drainage exudate have been shown to be enriched in MMPs including MMP-2 and MMP-9 [49]. However, since the focus of this study was to evaluate the EV surface markers and not soluble factors, we did not analyze the level of soluble EpCAM in the drainage fluid exudate.

Other cancer-related markers that were detected on the surface of the EVs from all patients were CD29, CD44 and CD146. These proteins are of special interest for the use as biomarkers, as well as for mechanistical studies, since they were detected in all patients. CD29 and CD146, two of the proteins that were detected at highest level among all proteins, were increased in patients with Her2 positive tumors compared with Her2 negative patients. However, since the number of patients included in this study is very small, this needs to be investigated further. In line with this finding, high CD29 (b1-integrin) expression has been associated with Her2 expression on tumor cells, while low expression of CD29 has been associated with Her2 negative tumors and a less aggressive phenotype [20]. Furthermore, the expression of integrins, including CD29, on EVs isolated from breast cancer cell lines, have been associated with tumor stage [55]. In a recent study it was demonstrated that breast cancer tissue had increased level of CD29 compared to healthy tissue [22]. CD146 has also been associated with aggressive cancers and poor prognosis [16]. To our knowledge, the expression of CD29 and CD146 on EVs isolated from breast cancer patients has not yet been evaluated. The investigation of CD29 and CD146 on EVs could help to increase the understanding of the role of EVs in cancer. However, it is important to stress that these findings have to be validated in larger cohorts of patients, where lymphatic exudate derived EVs can be compared to a proper control group. Healthy subjects would be the best control group, but ALND is not performed for any benign disease. Another option is to use lymphatic exudate from patients with other types of cancer, e.g. melanoma could potentially be of great interest. Future studies should also compare the findings in the exudate with plasma or serum, as a way to validate the findings, and open up for the use of plasma or serum as controls from healthy subjects.

The platelet markers CD41b and CD42a, and the activation marker CD62p, was present on EV isolations from all patients. Platelets are increased in different cancers including breast cancer and have a role in tumor growth and metastasis. Platelets release large amounts of EVs that may be important mediators in those events [56]. Interestingly, the platelet markers CD41b, CD42a and CD62p were increased in EVs from patients with large tumors, and the patient with recurrent disease, compared to patients with smaller tumors, indicating an increase in platelet-derived EVs in those patients.

Cancer stem cells (CSCs) are a population of cancer cells within a tumor that have the capacity to self-renew and differentiate to the diverse cells that comprise the tumor. They are also more resistant to chemotherapy which makes them important therapeutic targets for future therapies [57]. The combination of different markers including CD24, CD29, CD44, CD105 and CD133 have been suggested as markers for CSCs from different tumors [58–60]. CSCs from breast tumors are suggested to have high expression of CD44 and low or no expression of CD24 [23]. Furthermore, Her2 and CD44 positive EVs has been associated with tumor recurrence and metastasis [19]. Even though CSCs have been studied extensively the last 20 years, there is not yet much knowledge about CSC-derived EVs [44]. The lymphatic drainage EVs in this study harbor several of those CSC markers, including CD29, CD44 and CD105 and a low level of CD24, which suggests that a portion of the EVs might be of CSC origin.

## Conclusion

In this study, we show that lymphatic drainage exudate retrieved from patients with breast cancer after surgery contain EVs. Using SEC isolation, a large number of EVs were isolated and surface protein profiling revealed that the EVs contain several cancer-related markers, including CD29, CD44 and CD146. These proteins are of potential interest as biomarkers as well as to increase the understanding of the mechanisms of cancer biology, especially in the context of role of EVs in the development of lymph node metastasis. Two distinct clusters, seemingly related to tumor burden, was identified and EVs from Her2 positive patients were enriched in the cancer-related markers CD29 and CD146. These findings are of great interest and further investigation in larger patient materials are needed.

## Supplementary Information

**Additional file 1.** SEC isolation of EVs. Particle number and protein amount after SEC isolation of EVs and collection of 10 fractions from 1 patient. The particle number was quantified by NTA and protein amount by Qubit. The results suggests that most EVs are eluted in fraction 1–5, while proteins elute in fraction 6 and later.

**Additional file 2.** Information about particle and protein concentration as estimated by QUBIT and nFCM for each of the 7 patients. The table include information about Her2+ or Her2-, protein and particle quantification data for each patient.

**Additional file 3.** Quantification of EVs by NTA, nFCM and protein quantification. (A) Particle and protein quantification of EVs using NTA and Qubit. (B) Comparison of particle quantification by NTA and nFCM. Values are particles or proteins per ml of lymphatic drainage fluid from three 7 breast cancer patients.

**Additional file 4.** Size distribution and concentration of EVs quantified by nFCM. nFCM measurement of EVs from the seven breast cancer patients. The graphs shows the particle size (diameter) distribution and concentration per ml of lymphatic drainage fluid. Gating range: 25–125 nm. ≥99% of the particles analyzed were within the gating range.

**Additional file 5.** Detection of EV surface proteins using multiplex bead-based flow cytometry assay. Data is shown as background corrected (isotype control and blank samples) median fluorescence intensity (MFI) of all 37 markers for the 7 patients. The dashed line at MFI 20 indicates threshold for positive signal.

**Additional file 6.** Western blot for EpCAM. EpCAM was evaluated by western blot, confirming the absence of EpCAM in patient EV samples. BT-474 cells and EVs were used as positive control (2 μg BT-474 cell protein, 10 μg BT-474 EV and patient EV protein was loaded in each lane).

**Additional file 7.** Uncropped western blot images. Uncropped images of all western blots included in the manuscript are shown in the figure. For patient 3, 5 and 6, proteins extracted from individual SEC fractions (F1–7 or 8) were separated on the gel and blotted for GRP94, albumin, CD63, Flotillin-1 and ApoA1. Proteins extracted from patient 1–7 was analyzed for albumin, CD63, Flotillin-1, ApoA1 and CD146. In each lane, 10 μg of proteins (9 for patient 2) was loaded for pooled EV fraction 2–4 and 20 μg for individual fractions except for F1 in patient 4 and 6 where the maximum volume was loaded (2.5 and 3.4 μg respectively) due to low protein concentration in those samples. The positive control (CTR+) used was proteins extracted from MSC cell lysate (Grp94 and Flotillin-1), EVs from MSCs (CD63), human melanoma metastatic tissue (albumin and ApoA1). For CD146, the unstained gel is shown which was used for total protein normalization step.

## Data Availability

All data generated or analyzed during this study are included in this published article and its supplementary information files.

## Abbreviations

ALND: Axillary lymph node dissection
ApoA1: Apolipoprotein A1
BCS: Breast conserving surgery
ER: Endoplasmic reticulum
ER: Estrogen receptor
FNB: Fine needle biopsy
HER2: Human epidermal growth factor receptor 2
LVI: Lymphovascular invasion
NACT: Neoadjuvant chemotherapy
NHG: Nottingham histological grade
NTA: Nanoparticle tracking analysis
PR: Progesterone receptor
RT: Room temperature
SEC: Size exclusion chromatography
SNB: Sentinel node biopsy
