# Supplementary material for: Characterization of surface markers on extracellular vesicles isolated from lymphatic exudate from patients with breast cancer
Source: BMC Cancer. 2022 Jan 10;22:50. doi: 10.1186/s12885-021-08870-w (PMC8744234; doi:10.1186/s12885-021-08870-w)
Supplement: Supplementary file 1 — Additional file 1. SEC isolation of EVs. Particle number and protein amount after SEC isolation of EVs and collection of 10 fractions from 1 patient. The particle number was quantified by NTA and protein amount by Qubit. The results suggests that most EVs are eluted in fraction 1–5, while proteins elute in fraction 6 and later. [file 12885_2021_8870_MOESM1_ESM.pdf]

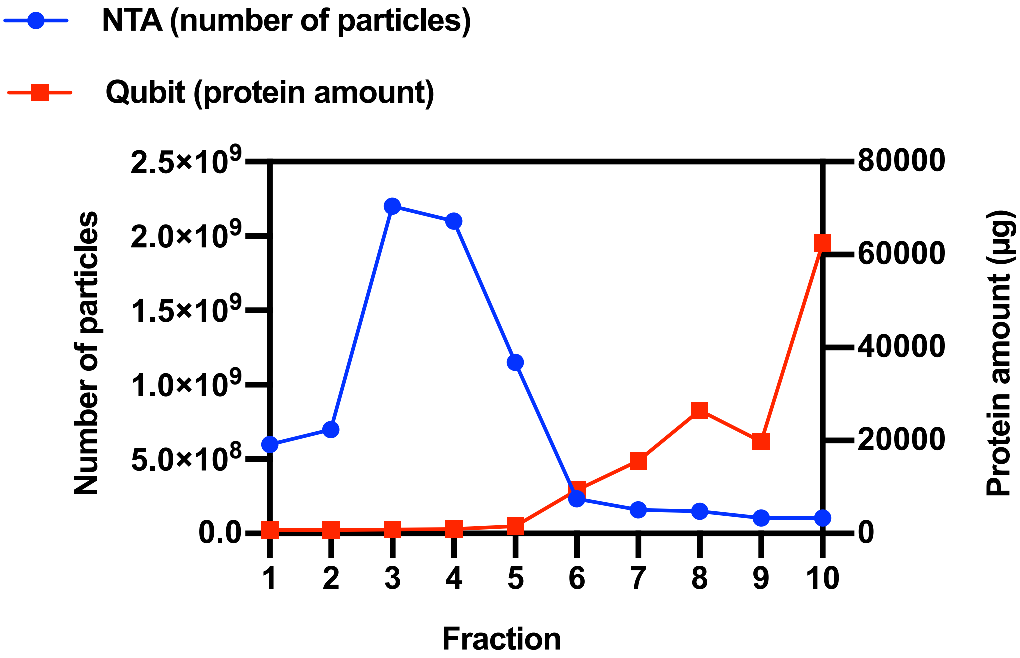

Additional file 1. Analysis of particle number and protein amount after SEC isolation. Ten SEC fractions were collected from one patient, and particle number and protein amount in each fraction was analysed to evaluate in which fractions EVs and non-EV proteins elute. The results suggests that most EVs are eluted in fraction 1-5, while proteins elute in fraction 6 and later.
