# Supplementary material for: Characterization of surface markers on extracellular vesicles isolated from lymphatic exudate from patients with breast cancer
Source: BMC Cancer. 2022 Jan 10;22:50. doi: 10.1186/s12885-021-08870-w (PMC8744234; doi:10.1186/s12885-021-08870-w)
Supplement: Supplementary file 2 — Additional file 2. Information about particle and protein concentration as estimated by QUBIT and nFCM for each of the 7 patients. The table include information about Her2+ or Her2-, protein and particle quantification data for each patient. [file 12885_2021_8870_MOESM2_ESM.pdf]

Patient number	Her2	Volyme lymphatic drainage fluid (ml)	Volume isolated EVs (ml)	Particle conc in isolated EV sample, nFCM (particles/µl)	Protein conc in isolated EV sample (µg/µl)	Protein amount in 5E8 particles (µg protein used for MACSPlex	Particle conc in lymphatic drainage fluid, nFCM (particles/ml)	Protein conc in lymphatic drainage fluid (µg/ml)
1	Negative	10	4.3	2.07E+07	0.44	11	8.90E+09	190
2	Negative	10	1.5	1.88E+07	0.27	7	2.82E+09	41
3	Positive	10	2.6	2.55E+07	0.47	9	6.63E+09	123
4	Positive	10	8.5	2.54E+07	0.54	11	2.16E+10	459
5	Negative	10	4.4	2.48E+07	0.47	10	1.09E+10	209
6	Positive	10	8	9.74E+06	0.57	29	7.79E+09	454
7	Negative	10	8	2.23E+07	0.61	14	1.78E+10	486

Additional file 2. Information about particle and protein concentration as estimated by Qubit and nFCM for each of the 7 patients.
