# Supplementary material for: Characterization of surface markers on extracellular vesicles isolated from lymphatic exudate from patients with breast cancer
Source: BMC Cancer. 2022 Jan 10;22:50. doi: 10.1186/s12885-021-08870-w (PMC8744234; doi:10.1186/s12885-021-08870-w)
Supplement: Supplementary file 3 — Additional file 3. Quantification of EVs by NTA, nFCM and protein quantification. (A) Particle and protein quantification of EVs using NTA and Qubit. (B) Comparison of particle quantification by NTA and nFCM. Values are particles or proteins per ml of lymphatic drainage fluid from three 7 breast cancer patients. [file 12885_2021_8870_MOESM3_ESM.pdf]

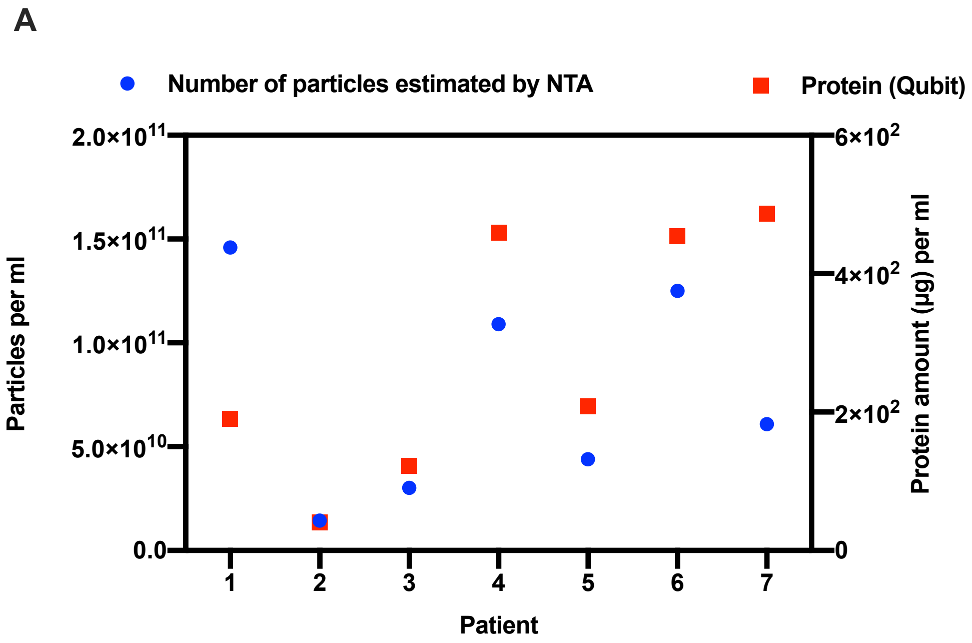

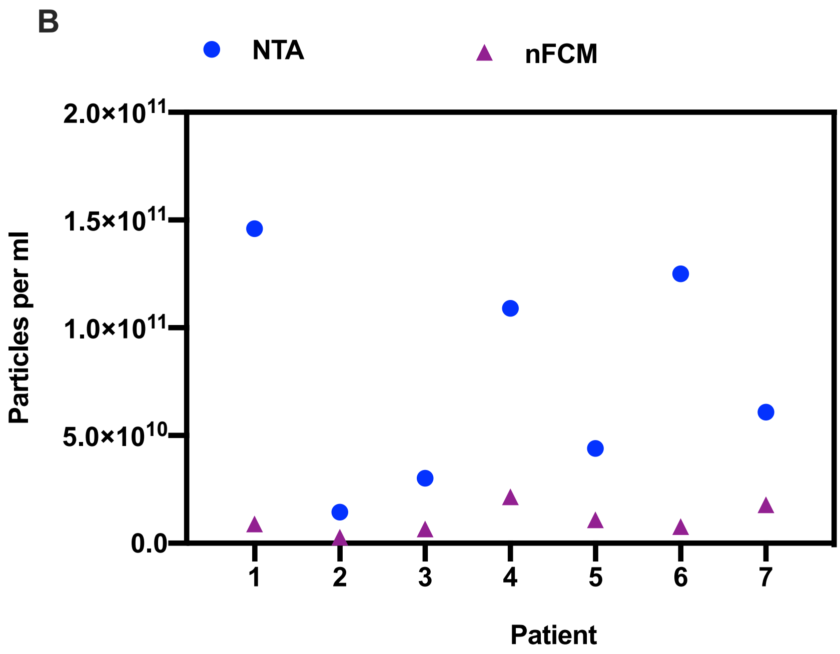

Additional file 3. Quantification of EVs by NTA, nFCM and protein quantification. (A) Particle and protein quantification of EVs using NTA and Qubit. (B) Comparison of particle quantification by NTA and nFCM. Values are particles or proteins per ml of lymphatic drainage fluid from three 7 breast cancer patients.
