# Supplementary material for: Characterization of surface markers on extracellular vesicles isolated from lymphatic exudate from patients with breast cancer
Source: BMC Cancer. 2022 Jan 10;22:50. doi: 10.1186/s12885-021-08870-w (PMC8744234; doi:10.1186/s12885-021-08870-w)
Supplement: Supplementary file 4 — Additional file 4. Size distribution and concentration of EVs quantified by nFCM. nFCM measurement of EVs from the seven breast cancer patients. The graphs shows the particle size (diameter) distribution and concentration per ml of lymphatic drainage fluid. Gating range: 25–125 nm. ≥99% of the particles analyzed were within the gating range. [file 12885_2021_8870_MOESM4_ESM.pdf]

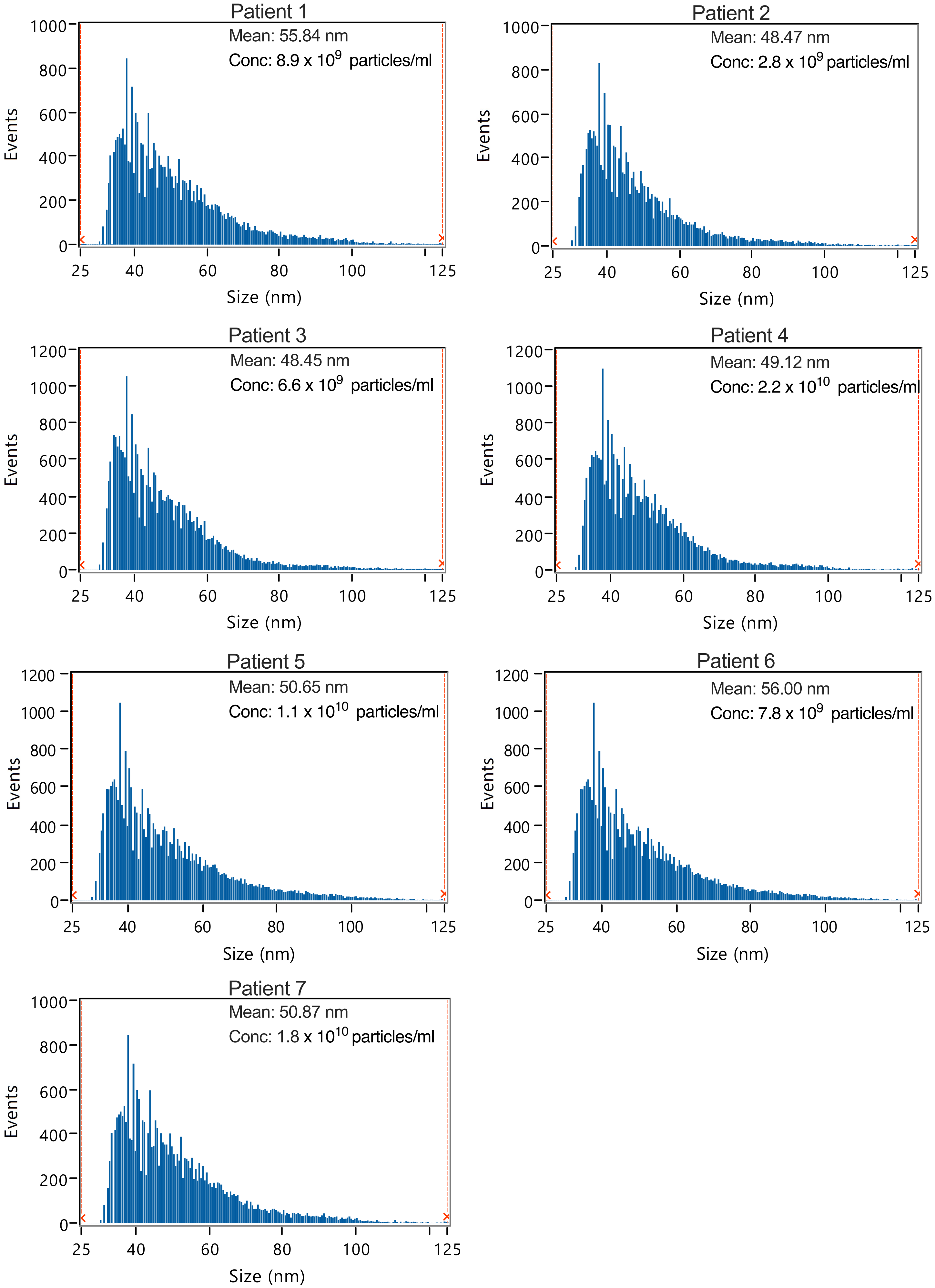

Additional file 4. Nano-Flow cytometry (nFCM) measurement of the particle size (diameter) distribution and concentration of EV preparationsfrom the 7 patients with breast cancer. Concentration values reported in the graph is back-calculated values for particles per ml of lymphatic drainage fluid. Gating range: 25-125 nm. ≥99% of the particles analysed were within the gating range.
