# Supplementary material for: Characterization of surface markers on extracellular vesicles isolated from lymphatic exudate from patients with breast cancer
Source: BMC Cancer. 2022 Jan 10;22:50. doi: 10.1186/s12885-021-08870-w (PMC8744234; doi:10.1186/s12885-021-08870-w)
Supplement: Supplementary file 5 — Additional file 5. Detection of EV surface proteins using multiplex bead-based flow cytometry assay. Data is shown as background corrected (isotype control and blank samples) median fluorescence intensity (MFI) of all 37 markers for the 7 patients. The dashed line at MFI 20 indicates threshold for positive signal. [file 12885_2021_8870_MOESM5_ESM.pdf]

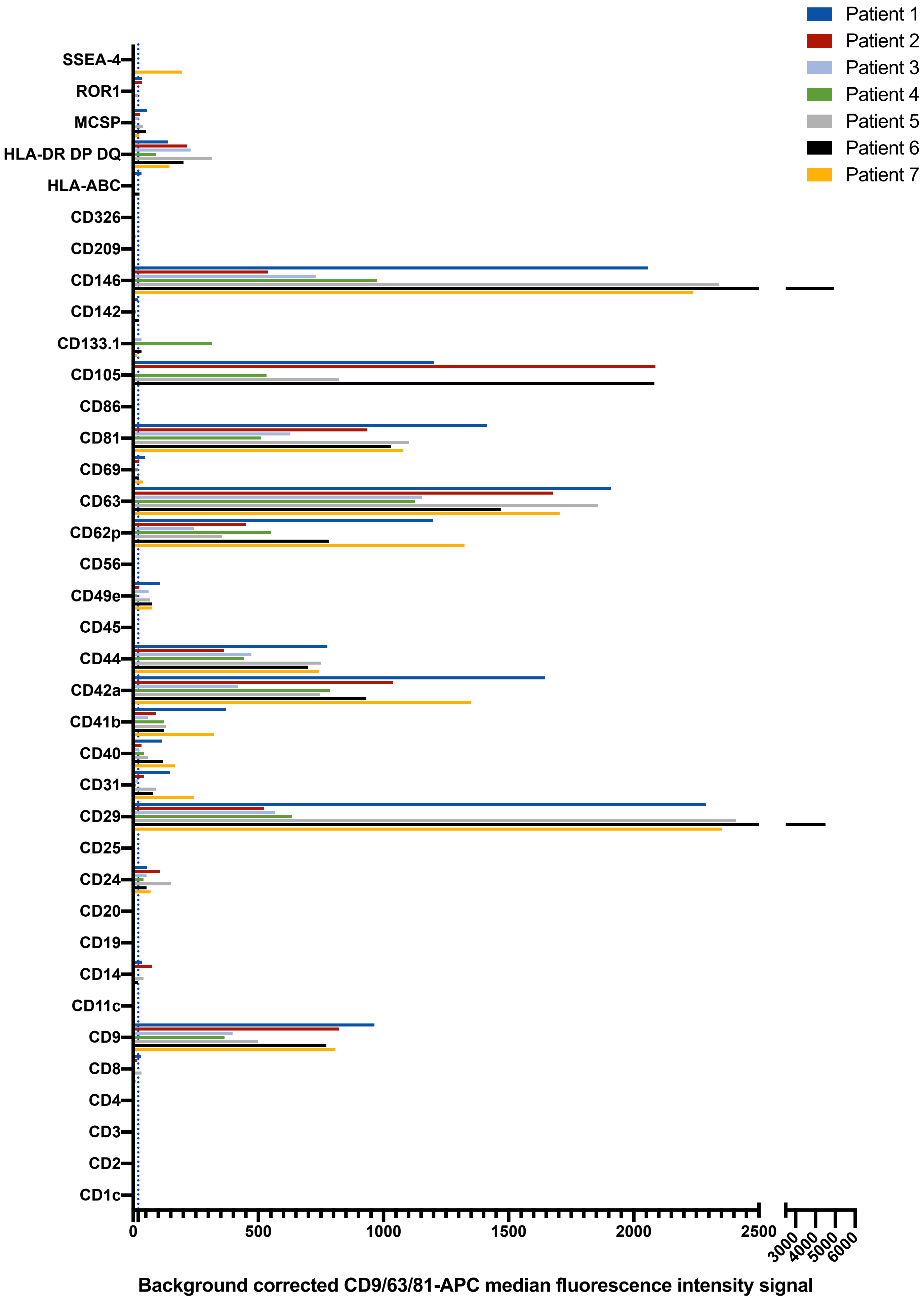

Additional file 5. Detection of EV surface proteins using multiplex bead-based flow cytometry assay. Data is shown as background corrected (isotype control and blank samples) median fluorescence intensity (MFI) of all 37 markers for the 7 patients. The dashed line at MFI 20 indicates threshold for positive signal.
