# Supplementary material for: Characterization of surface markers on extracellular vesicles isolated from lymphatic exudate from patients with breast cancer
Source: BMC Cancer. 2022 Jan 10;22:50. doi: 10.1186/s12885-021-08870-w (PMC8744234; doi:10.1186/s12885-021-08870-w)
Supplement: Supplementary file 6 — Additional file 6. Western blot for EpCAM. EpCAM was evaluated by western blot, confirming the absence of EpCAM in patient EV samples. BT-474 cells and EVs were used as positive control (2 μg BT-474 cell protein, 10 μg BT-474 EV and patient EV protein was loaded in each lane). [file 12885_2021_8870_MOESM6_ESM.pdf]

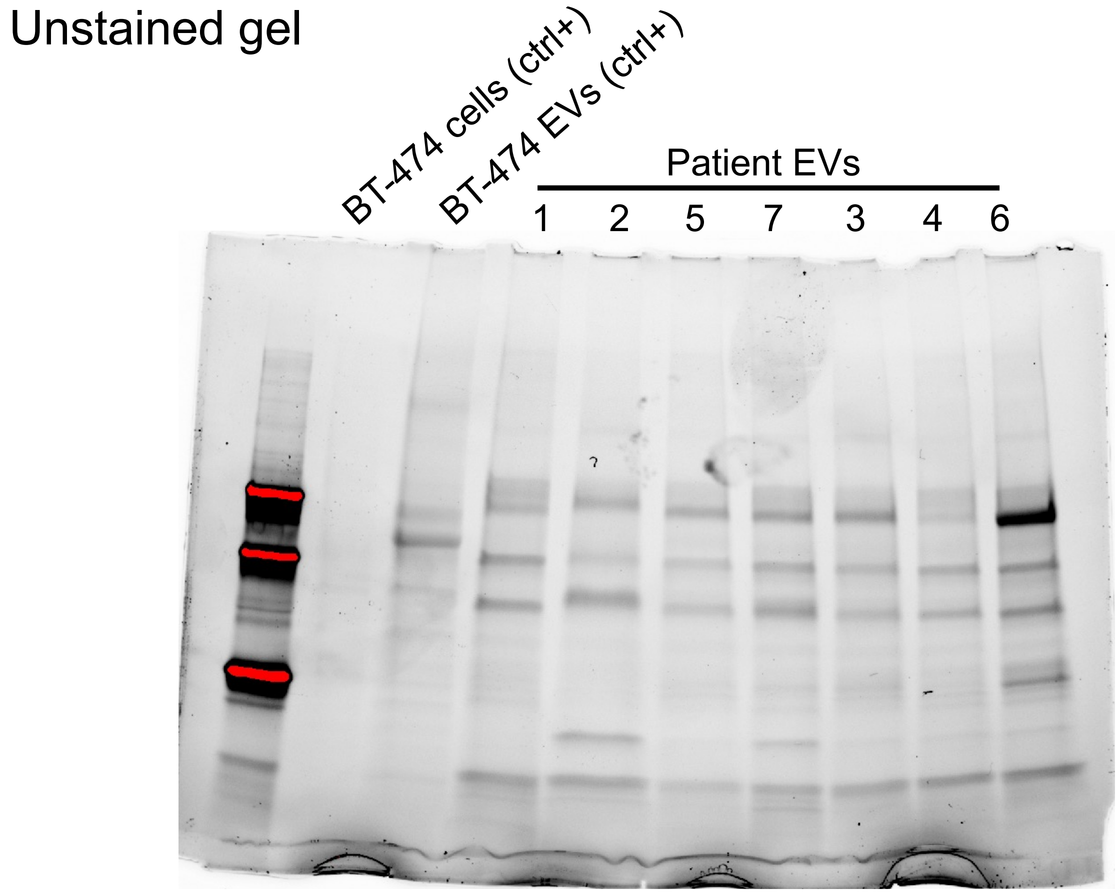

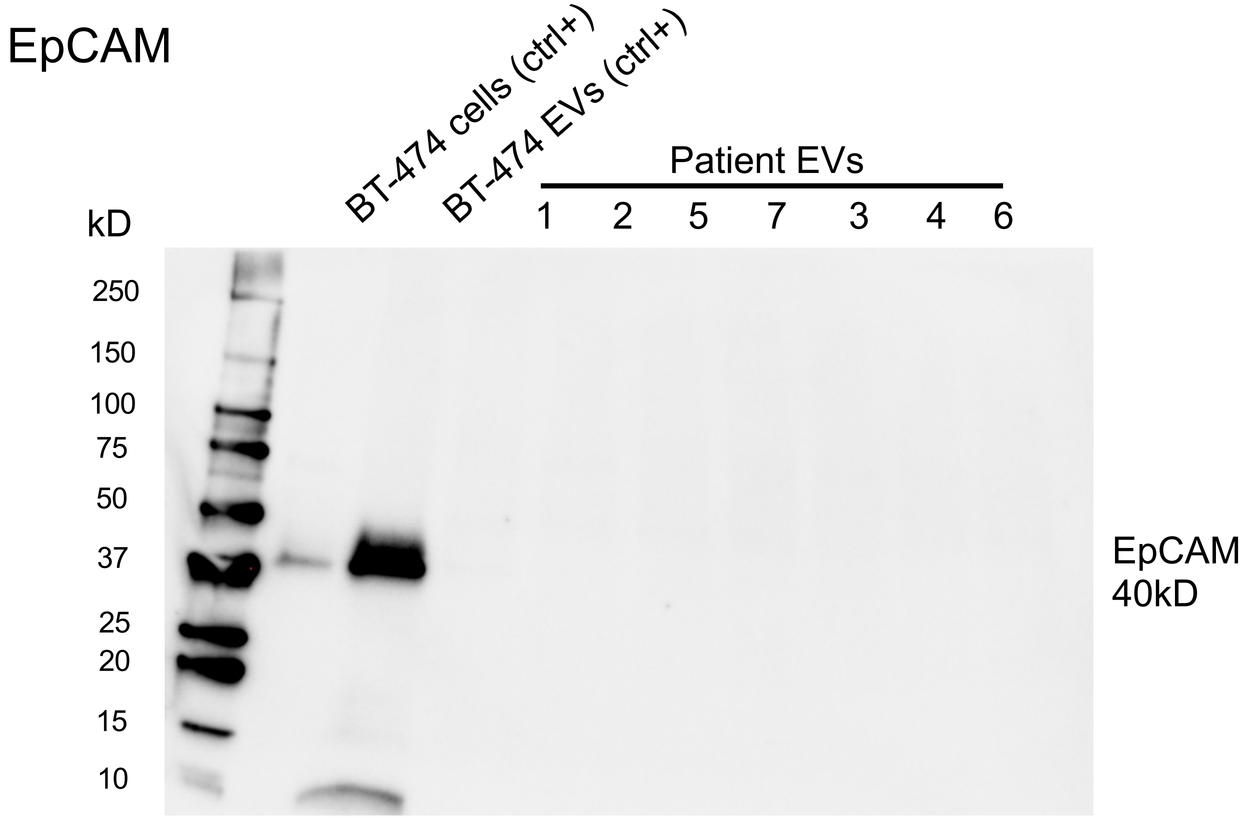

Additional file 6. Western blot for EpCAM. EpCAM was evaluated by western blot, confirming the absence of EpCAM in patient EV samples. BT-474 cells and EVs were used as positive control (2 µg BT-474 cell protein, 10 µg BT-474 EV and patient EV protein was loaded in each lane).
