# Supplementary material for: Characterization of surface markers on extracellular vesicles isolated from lymphatic exudate from patients with breast cancer
Source: BMC Cancer. 2022 Jan 10;22:50. doi: 10.1186/s12885-021-08870-w (PMC8744234; doi:10.1186/s12885-021-08870-w)
Supplement: Supplementary file 7 — Additional file 7. Uncropped western blot images. Uncropped images of all western blots included in the manuscript are shown in the figure. For patient 3, 5 and 6, proteins extracted from individual SEC fractions (F1–7 or 8) were separated on the gel and blotted for GRP94, albumin, CD63, Flotillin-1 and ApoA1. Proteins extracted from patient 1–7 was analyzed for albumin, CD63, Flotillin-1, ApoA1 and CD146. In each lane, 10 μg of proteins (9 for patient 2) was loaded for pooled EV fraction 2–4 and 20 μg for individual fractions except for F1 in patient 4 and 6 where the maximum volume was loaded (2.5 and 3.4 μg respectively) due to low protein concentration in those samples. The positive control (CTR+) used was proteins extracted from MSC cell lysate (Grp94 and Flotillin-1), EVs from MSCs (CD63), human melanoma metastatic tissue (albumin and ApoA1). For CD146, the unstained gel is shown which was used for total protein normalization step. [file 12885_2021_8870_MOESM7_ESM.pdf]

Patient 3

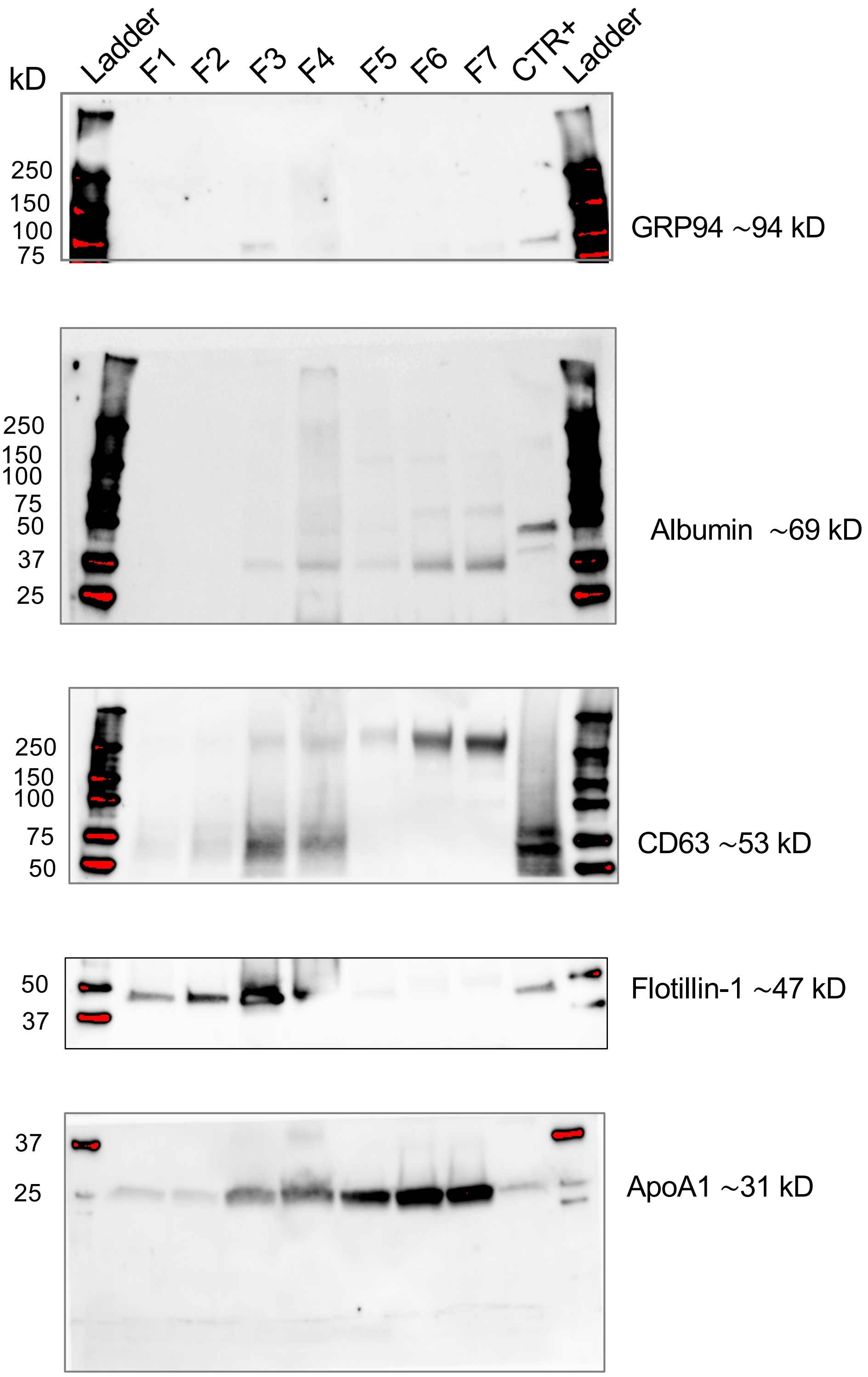
kD
Ladder
F1
F2
F3
F4
F5
F6
F7
CTR+
Ladder
250
150
100
75
GRP94 ~94 kD
250
150
100
75
50
37
25
Albumin ~69 kD
250
150
100
75
50
CD63 ~53 kD
50
37
Flotillin-1 ~47 kD
37
25
ApoA1 ~31 kD

Patient 5

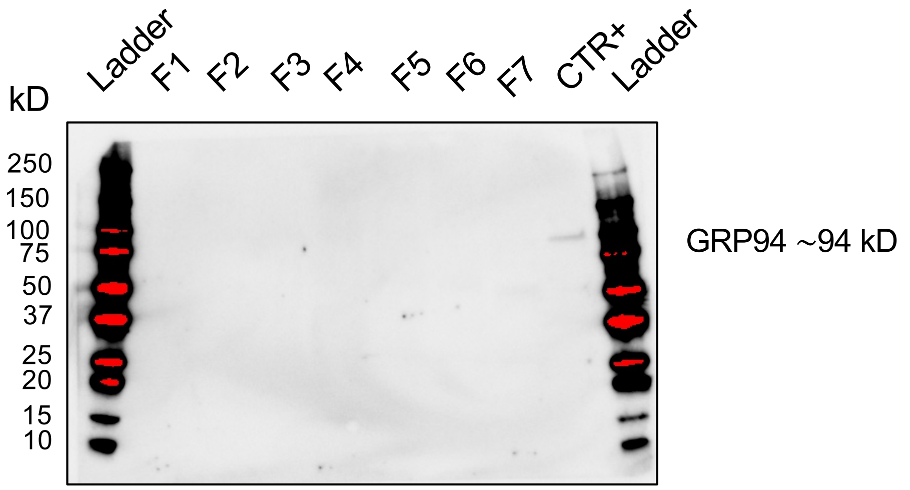
Ladder
F1
F2
F3
F4
F5
F6
F7
CTR+
Ladder
kD
250
150
100
75
50
37
25
20
15
10
GRP94 ~94 kD

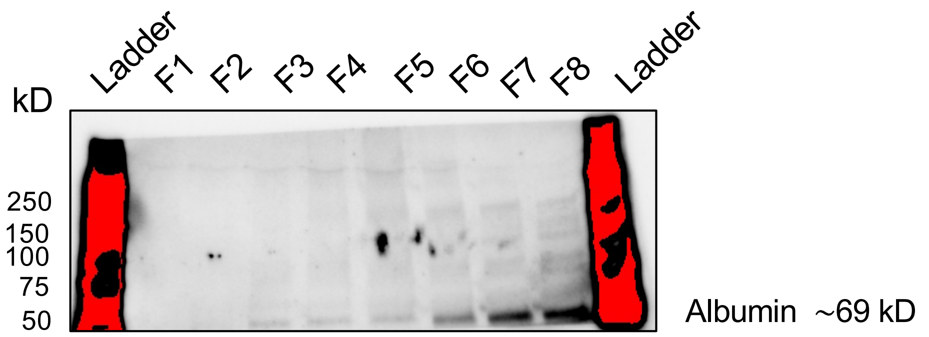
Ladder
F1
F2
F3
F4
F5
F6
F7
F8
Ladder
kD
250
150
100
75
50
Albumin ~69 kD

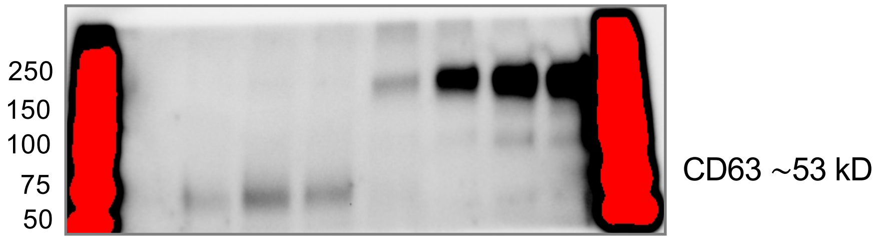
250
150
100
75
50
CD63 ~53 kD

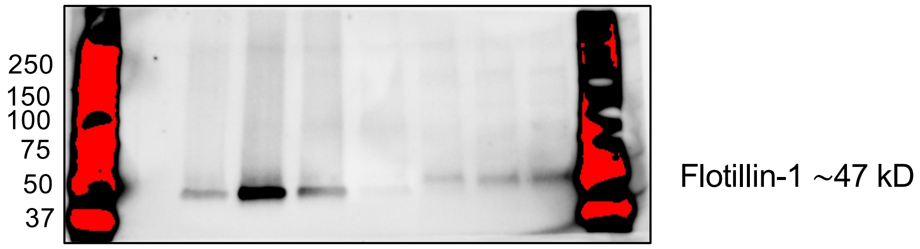
250
150
100
75
50
37
Flotillin-1 ~47 kD

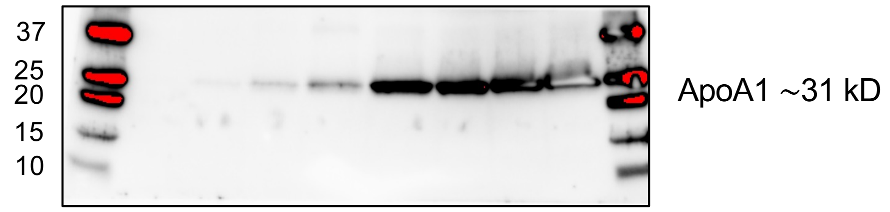
37
25
20
15
10
ApoA1 ~31 kD

Patient 6

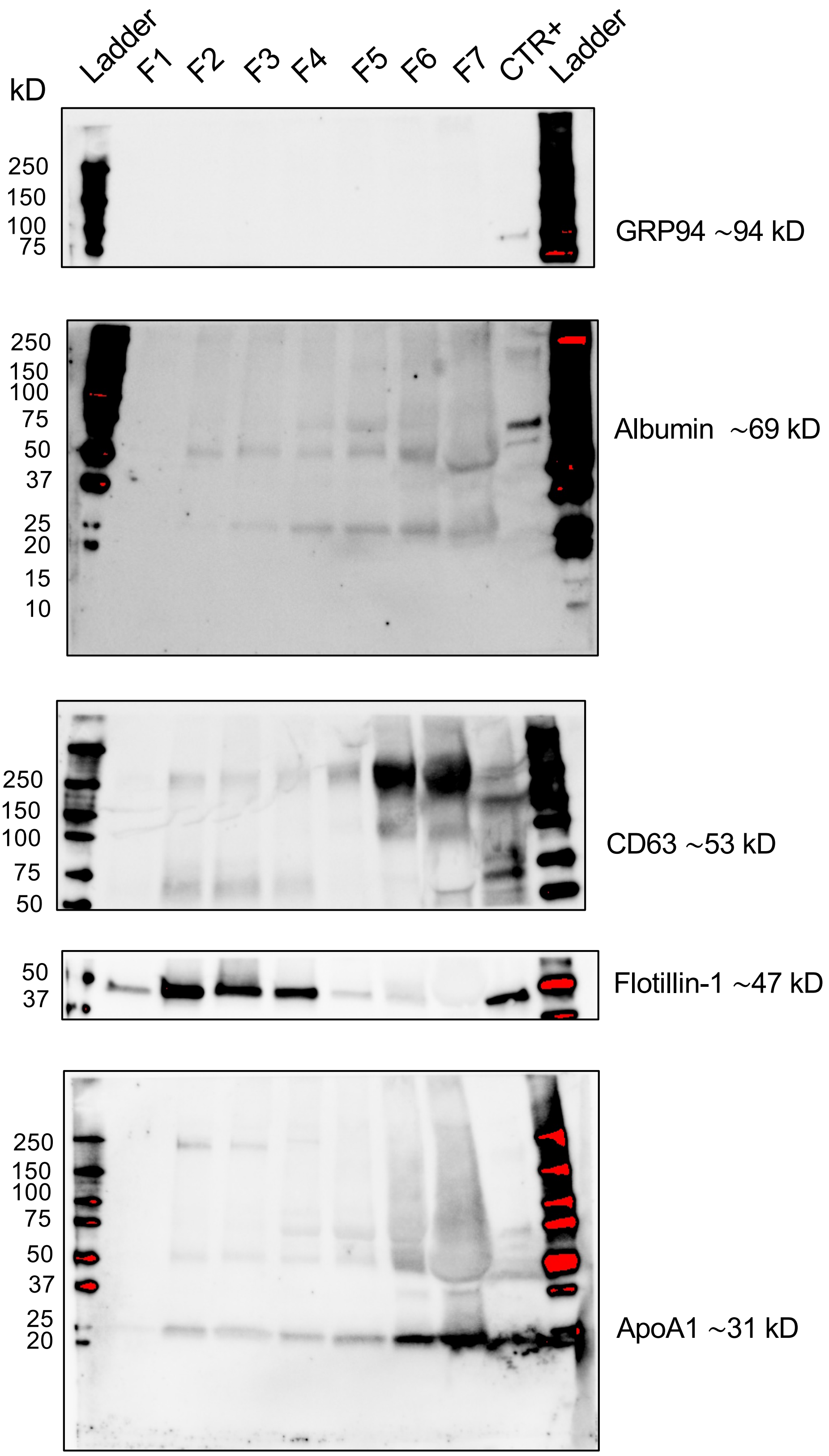
Ladder
F1
F2
F3
F4
F5
F6
F7
CTR+
Ladder
kD
250
150
100
75
GRP94 ~94 kD
250
150
100
75
50
37
25
20
15
10
Albumin ~69 kD
250
150
100
75
50
CD63 ~53 kD
50
37
Flotillin-1 ~47 kD
250
150
100
75
50
37
25
20
ApoA1 ~31 kD

All patients – fractions 2 - 4 combined

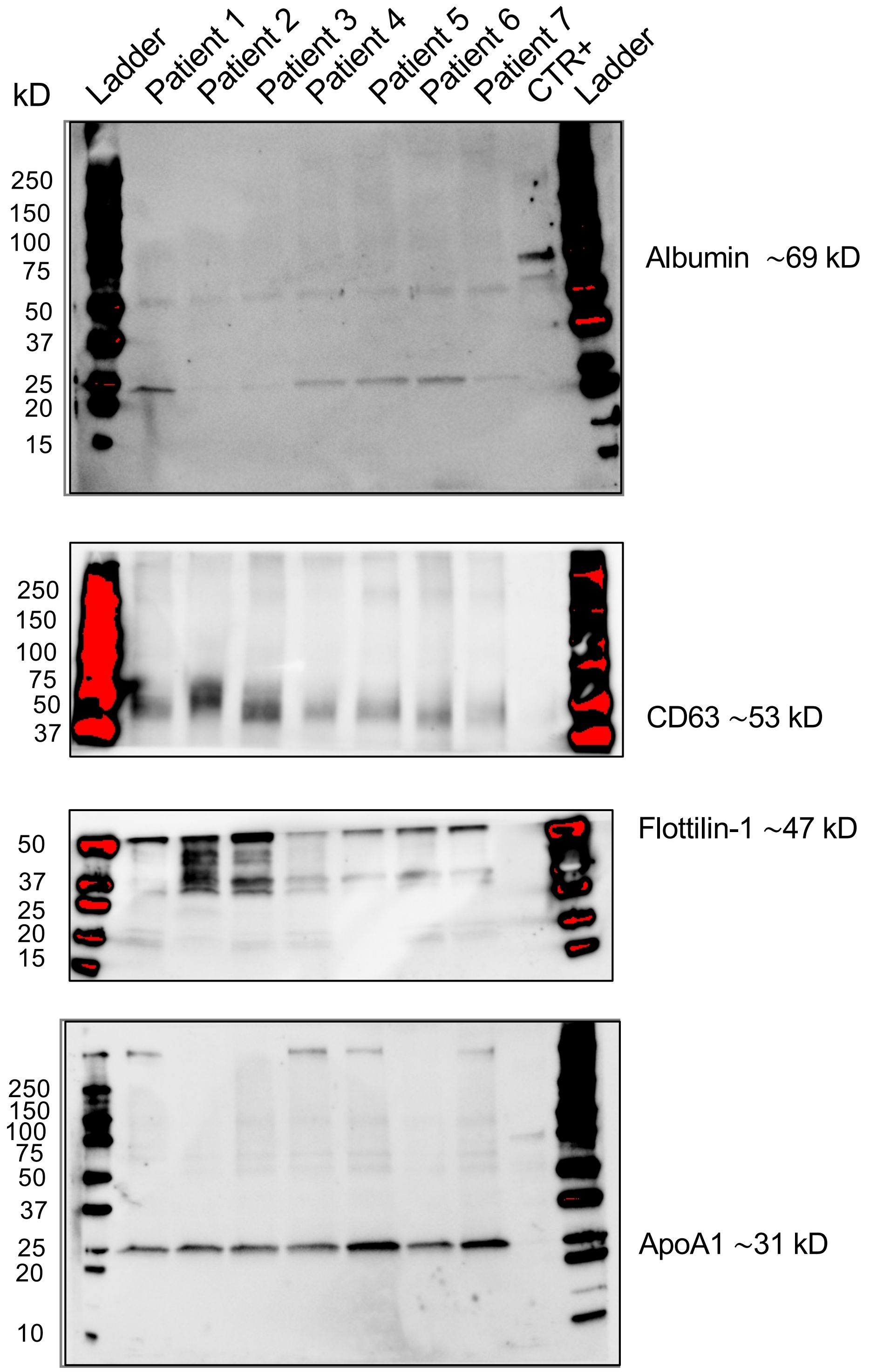

All patients – fractions 2 - 4 combined

Stain free gel, used for total protein normalization

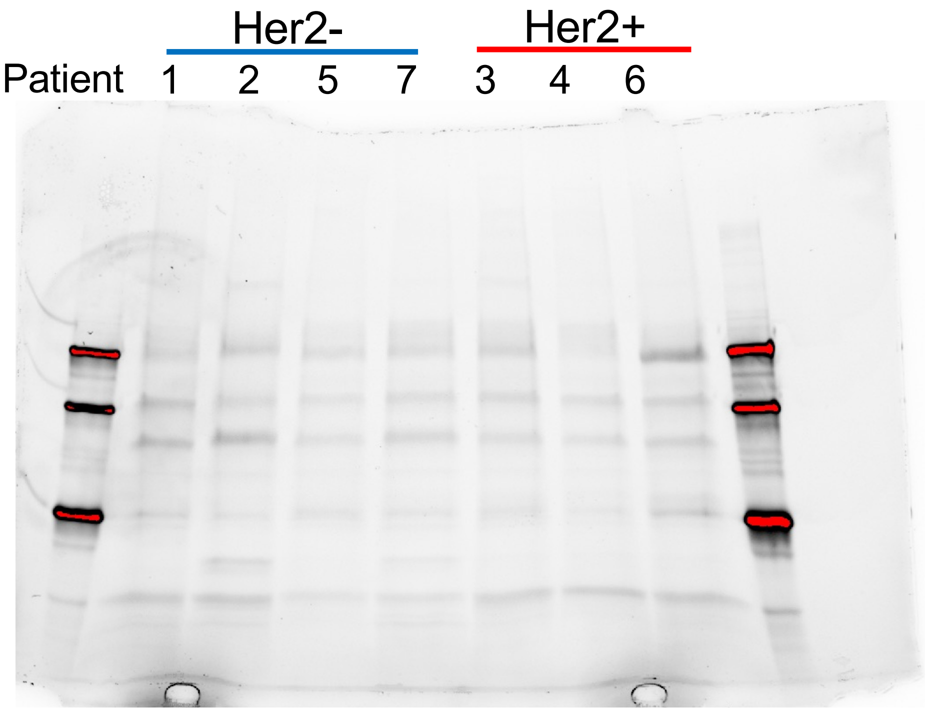

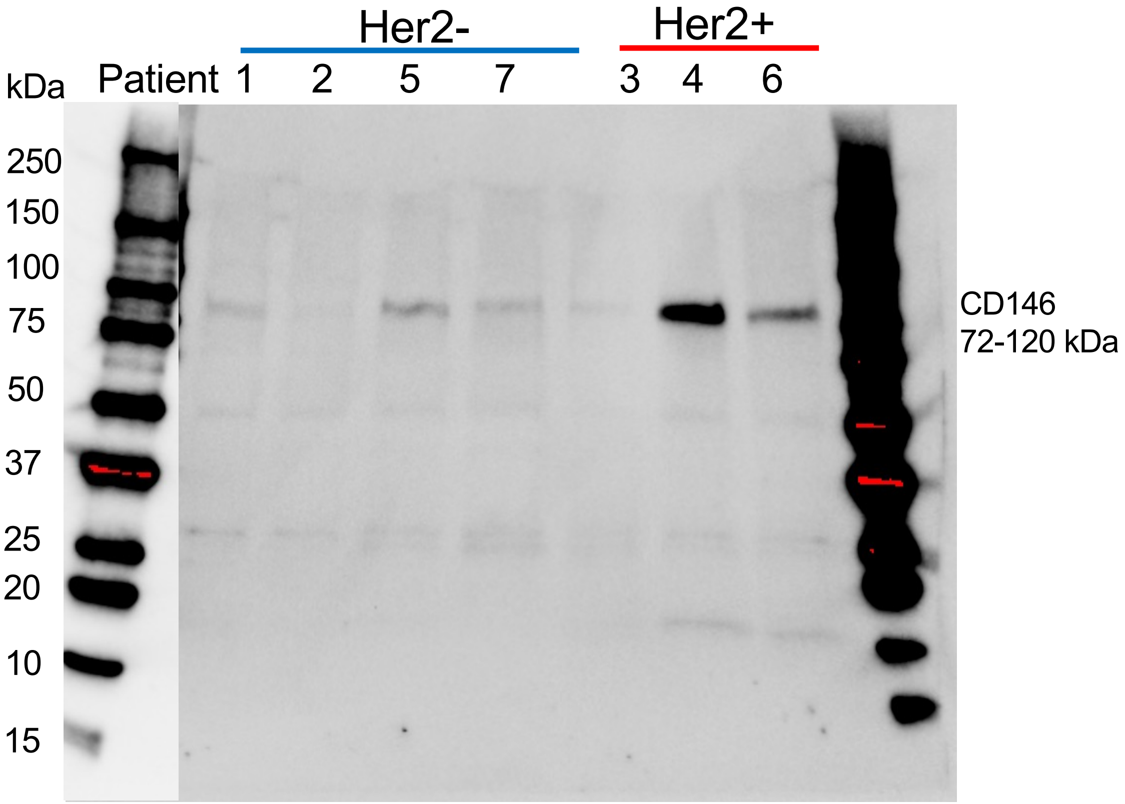

Additional file 7 . Uncropped images of all western blots included in the manuscript are shown in the figure. For patient 3, 5 and 6, proteins extracted from individual SEC fractions (F1-7 or 8) were separated on the gel and blotted for GRP94, albumin, CD63, Flotillin-1 and ApoA1. Proteins extracted from patient 1-7 was analyzed for albumin, CD63, Flotillin-1, ApoA1 and CD146. In each lane, 10 µg of proteins (9 for patient 2) was loaded for pooled EV fraction 2-4 and 20 µg for individual fractions except for F1 in patient 4 and 6 where the maximum volume was loaded (2.5 and 3.4 µg respectively) due to low protein concentration in those samples. The positive control (CTR+) used was proteins extracted from MSC cell lysate (Grp94 and Flotillin-1), EVs from MSCs (CD63), human melanoma metastatic tissue (albumin and ApoA1). For CD146, the unstained gel is shown which was used for total protein normalization step.
